# Core–shell nanoparticles suppress metastasis and modify the tumour-supportive activity of cancer-associated fibroblasts

**DOI:** 10.1186/s12951-020-0576-x

**Published:** 2020-01-21

**Authors:** Dávid Kovács, Nóra Igaz, Annamária Marton, Andrea Rónavári, Péter Bélteky, László Bodai, Gabriella Spengler, László Tiszlavicz, Zsolt Rázga, Péter Hegyi, Csaba Vizler, Imre M. Boros, Zoltán Kónya, Mónika Kiricsi

**Affiliations:** 1 Department of Biochemistry and Molecular Biology, Faculty of Science and Informatics, University of Szeged, Közép fasor 52, 6726 Szeged, Hungary; 2 Doctoral School of Biology, University of Szeged, Közép fasor 52, 6726 Szeged, Hungary; 3 Institute of Biochemistry, Biological Research Center of the Hungarian Academy of Sciences, Temesvári Krt. 62, 6726 Szeged, Hungary; 4 Interdisciplinary Excellence Centre, Department of Applied and Environmental Chemistry, University of Szeged, Rerrich B. tér 1, 6720 Szeged, Hungary; 5 Department of Medical Microbiology and Immunobiology, Faculty of Medicine, University of Szeged, Dóm tér 9, 6720 Szeged, Hungary; 6 Department of Pathology, University of Szeged, Állomás u. 2, 6725 Szeged, Hungary; 7 First Department of Medicine, University of Szeged, Korányi fasor 8-10, 6720 Szeged, Hungary; 8 MTA-SZTE Lendület Translational Gastroenterology Research Group, Korányi fasor 8-10, 6720 Szeged, Hungary; 9 MTA-SZTE Reaction Kinetics and Surface Chemistry Research Group, Rerrich B. tér 1, 6720 Szeged, Hungary

**Keywords:** Core–shell nanoparticles, Tumour stroma, Cancer-associated fibroblasts, Metastasis, RNA sequencing

## Abstract

**Background:**

Although accumulating evidence suggests that the crosstalk between malignant cells and cancer-associated fibroblasts (CAFs) actively contributes to tumour growth and metastatic dissemination, therapeutic strategies targeting tumour stroma are still not common in the clinical practice. Metal-based nanomaterials have been shown to exert excellent cytotoxic and anti-cancerous activities, however, their effects on the reactive stroma have never been investigated in details. Thus, using feasible in vitro and in vivo systems to model tumour microenvironment, we tested whether the presence of gold, silver or gold-core silver-shell nanoparticles exerts anti-tumour and metastasis suppressing activities by influencing the tumour-supporting activity of stromal fibroblasts.

**Results:**

We found that the presence of gold-core silver-shell hybrid nanomaterials in the tumour microenvironment attenuated the tumour cell-promoting behaviour of CAFs, and this phenomenon led to a prominent attenuation of metastatic dissemination in vivo as well. Mechanistically, transcriptome analysis on tumour-promoting CAFs revealed that silver-based nanomaterials trigger expressional changes in genes related to cancer invasion and tumour metastasis.

**Conclusions:**

Here we report that metal nanoparticles can influence the cancer-promoting activity of tumour stroma by affecting the gene expressional and secretory profiles of stromal fibroblasts and thereby altering their intrinsic crosstalk with malignant cells. This potential of metal nanomaterials should be exploited in multimodal treatment approaches and translated into improved therapeutic outcomes.

## Background

Metastasis is considered as the most fatal hallmark of cancer, and the poor therapeutic outcome of patients diagnosed with invasive tumours illustrates its true gravity. Although many novel therapeutic strategies have recently been developed aiming to selectively kill malignant cells, accumulating evidence indicates that not only cancerous cells, but the cellular components of the reactive stroma should be targeted as well [1, 2]. The cellular composition of the tumour stroma is largely heterogeneous; fibroblasts, macrophages and other immune cells are the most frequent cancer-associated cell types in the tumour microenvironment [3]. Formation of a cancer-supporting milieu involves the recruitment and concomitant conversion of various stromal cells into cancer-favouring phenotypes via intensive reciprocal crosstalk employing a multitude of secreted factors [4]. The most abundant non-cancerous cell population within the reactive stroma is of cancer-associated fibroblast (CAF) as they might represent the 80% of the total tumour mass in pancreatic tumours [5]. The cancer-promoting activity of CAFs has been relatively well-characterised, and it has been demonstrated that they interact with cancer cells during all stages of tumour development [6, 7]. The continuous and mutual information exchange between CAFs and cancer cells supports the generation of the pre-metastatic niche, to which CAFs contribute primarily by secreting a plethora of growth factors, releasing tumour-stimulating exosomes, inducing epithelial-to-mesenchymal transition and neo-angiogenesis, and by remodelling the components of the extracellular matrix [8–11]. Moreover, it has already been shown, that via various mechanisms, CAFs can promote the evolution of a multidrug-resistant tumour phenotype [12]. Many studies reported that pharmacological modification of the crosstalk between CAFs and tumour cells can hamper metastasis and ameliorate survival, further highlighting the supportive function of CAFs upon metastatic dissemination [13–15].

In addition to small-molecular anti-cancer compounds, nano-sized materials represent promising alternatives in the development of novel therapeutic modalities [16]. Based on the unique physicochemical and biological properties of nanoparticle systems, and owing to the simple and economical synthesis, the potential of metal nanoparticles in medicine is intensively investigated [17]. Various studies on in vivo and in vitro cancer models have reported that metal nanoparticles made of gold, silver, platinum, titanium or copper can effectively suppress tumour growth mainly by inducing apoptosis in cancer cells [18–21]. As an example, silver nanoparticle (AgNP) action on cancer cells—often referred to as a “Trojan-horse” type mechanism—encompasses cellular nanoparticle uptake, the subsequent release of reactive ions and generation of a vast amount of reactive oxygen species, which ultimately target cellular macromolecules and organelles, altogether leading to the initiation of the apoptotic process [22]. Although this effect can be exploited in cancer treatment, it can also lead to systemic toxicity in non-target organs [23, 24]. In contrast, gold nanoparticles (AuNP) are considered to be relatively non-reactive and fairly biocompatible. Due to some singular physico-chemical characteristics, AuNPs have also been studied for therapeutic and diagnostic purposes [25]. Since they are capable of elevated photoelectric absorption of radiation energy compared to soft tissues, these nanomaterials are recognised as excellent enhancers of radiotherapy [26]. In addition, gold nanoparticles are outstanding delivery platforms of cytotoxic drugs or therapeutic genes, which can ultimately be directed—rather selectively—into the microenvironment of the cancerous tissues, by exploiting the characteristic vascular abnormalities of solid tumours, often cited as enhanced permeability and retention effect (EPR) [27].

Although the direct impact of metallic nanoparticles on cancer cells is well-characterised, yet it is unknown how such nanoparticles would affect the reactive stroma, and in particular CAFs. Therefore, in this study, we investigated the behaviour and the tumour-promoting functions of CAFs upon metal nanoparticle exposures. In order to exploit the anti-cancer features of nanosilver, but at the same time to attenuate its systemic toxicity, as well as to profit from the biocompatible nature of gold nanoparticles, we generated a gold-core silver-shell-structured hybrid nanoparticle system (Au@Ag) and tested their in vitro and in vivo performance, besides that of AgNPs and AuNPs. Our primary goal was to dissect the differences in biological effects exhibited by gold-core silver-shell nanoparticles with those of only silver containing nanoparticles of the same size as Au@Ag, and secondly, to analyse the cellular response to AuNPs in order to model the potential impact of the gold core of the Au@Ag. Our broader aim was to explore, whether deploying gold- and silver-based nanoparticles in the tumour microenvironment would influence the crosstalk between fibroblasts and cancer cells in such a way to improve tumour therapy outcome.

## Results

### AgNP and Au@Ag nanoparticles selectively inhibit adenocarcinoma cells

In order to obtain gold-core silver-shell nanoparticles, first, AuNPs had to be prepared using a chemical reduction method. The successful synthesis of AuNPs was verified by TEM and UV–Vis analysis (Additional file 1). The hydrodynamic diameter of the obtained AuNPs was approximately 8–9 nm. These AuNPs were used as seeds for the preparation of the citrate-stabilised gold-core silver-shell Au@Ag nanoparticles, by establishing a covering silver layer. The larger size of Au@Ag (approx. 11 nm) nanoparticles compared to AuNPs detected by DLS and TEM, and UV–Vis spectra characteristic to silver nanoparticles all indicated the successful synthesis of hybrid nanoparticles and verified the silver coverage of the gold surface. Finally, since we aimed to compare the performance of these hybrid nanoparticles with those of AgNPs, citrate-stabilised silver nanoparticles of the same size as Au@Ag nanoparticles (approx. 12 nm) were synthesised and subsequently characterised (Additional file 1).

**Fig. 1:**
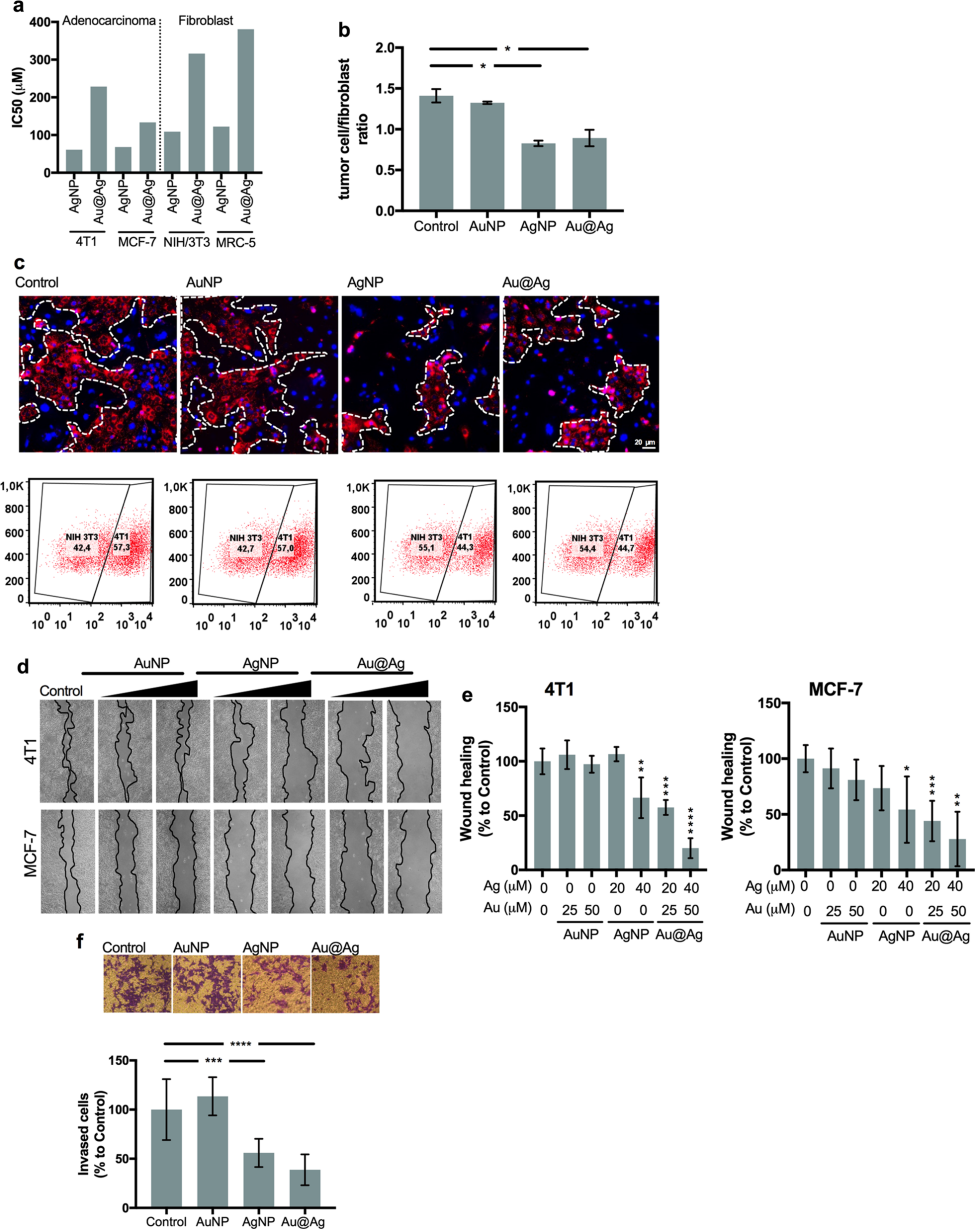
AgNP and Au@Ag nanoparticles selectively inhibit breast adenocarcinoma cells. Breast cancer cells (4T1, MCF-7) and fibroblast cells (NIH/3T3, MRC-5) were treated with AgNP and Au@Ag nanoparticles, respectively, for 24 h then IC_50_ values were calculated. AgNPs exerted higher cytotoxicity than Au@Ag nanoparticles, moreover, fibroblast cells were less sensitive to nanoparticle treatments than cancer cells (**a**). Fluorescently labelled 4T1 tumour cells were co-cultured with unlabelled NIH/3T3 fibroblasts then treated with nanoparticles in non-toxic concentrations. Microscopic images (**c**) and flow cytometric evaluation of the co-cultures (**b**, **c**) revealed that AgNP and Au@Ag nanoparticle treatments reduced the ratio of cancerous cells in the tested co-cultures, while AuNP treatments did not influence the cellular composition of the samples (**b**, **c**). AgNP and Au@Ag nanoparticle treatments suppressed the wound healing activity of 4T1 and MCF-7 adenocarcinoma cells (**d**, **e**) demonstrated by cell free zones 24 h after scratching (**d**) and by quantitative evaluation of the wound healing activities (**e**). AgNP and Au@Ag nanoparticle treatments suppressed also the invasion of 4T1 adenocarcinoma cells (**f**). *P ≤ 0.05; **P ≤ 0.01; ***P ≤ 0.001; ****P ≤ 0.0001 indicate statistical significance (unpaired *t*-test)

To assess the cytotoxicity of the as-prepared silver-containing nanoparticles, surviving curves and their corresponding IC_50_ values were determined upon 24 h AgNP and Au@Ag treatments on various adenocarcinoma and fibroblast cell lines (Additional file 2a and Fig. 1a). Our results demonstrate, that Au@Ag nanoparticles are considerably less toxic than AgNPs to all the applied cell lines. Furthermore, the obtained IC_50_ values indicate that 4T1 and MCF-7 adenocarcinoma cells are more sensitive to both AgNP and Au@Ag nanoparticle treatments than non-cancerous NIH/3T3 and MRC-5 fibroblast cells. The cytotoxicity of the AuNP nanoparticles was also tested, however, on tumour cells no toxicity and on NIH/3T3 and MRC-5 fibroblast cells only minor cytotoxicity was detected (Additional file 2b). To further examine the impact of metal nanoparticles on the behaviour of healthy fibroblasts, wound healing experiments were performed on NIH/3T3 and MRC-5 fibroblasts and their migration capacities were tested. None of the nanoparticle treatments reduced the number of migrating fibroblasts (Additional file 3).

To mimic the milieu of the reactive stroma, fluorescently-labelled 4T1 adenocarcinoma cells were co-cultured with non-labelled NIH/3T3 fibroblasts, then co-cultures were treated with AgNP or Au@Ag nanoparticles in non-toxic concentrations (20 μM silver) or with AuNPs in concentrations corresponding to the amount of gold that the cells would receive over Au@Ag nanoparticle treatments (25 μM gold). When co-cultures were exposed to either AgNP or Au@Ag nanoparticles, smaller tumour cell islands were observed by fluorescent microscopy compared to untreated co-cultures. Furthermore, flow cytometry revealed that the tumour cell/fibroblast ratio also decreased upon these nanoparticle treatments. On the other hand, gold nanoparticles did not influence the composition of the co-cultures (Fig. 1b, c).

Wound healing assays were performed on 4T1 and MCF-7 adenocarcinoma cells to assess the influence of metal-nanoparticles on cancer cell migration. While AuNPs did not inhibit wound closure, AgNP and Au@Ag nanoparticles exhibited significant inhibitory action on both 4T1 and MCF-7 cells (Fig. 1d, e). Additionally, to ensure that the observed suppression in wound closure was not the result of activated apoptotic or necrotic pathways, cells were collected following wound healing assays, stained with Annexin V/Propidium iodide and the percentages of apoptotic and necrotic cells were quantified. The well-characterised apoptosis inducer 12H-benzo[alpha]phenothiazine (M627) was used as a positive control (Additional file 4). None of the nanoparticle treatments triggered either apoptotic or necrotic cell death, indicating that the observed migration-suppressing activity of Au@Ag and AgNPs is not coupled to cytotoxicity.

Finally, to test the potential inhibitory effect of the metal nanoparticle treatments on cell invasion, transmigration capability of invasive 4T1 cells was investigated using Boyden chamber experiments. Gold nanoparticles did not suppress the invasion of 4T1 cells, while a significantly reduced cell invasion was observed upon AgNP or Au@Ag nanoparticle exposures (Fig. 1f).

### Au@Ag nanoparticles inhibit metastasis in vivo

**Fig. 2:**
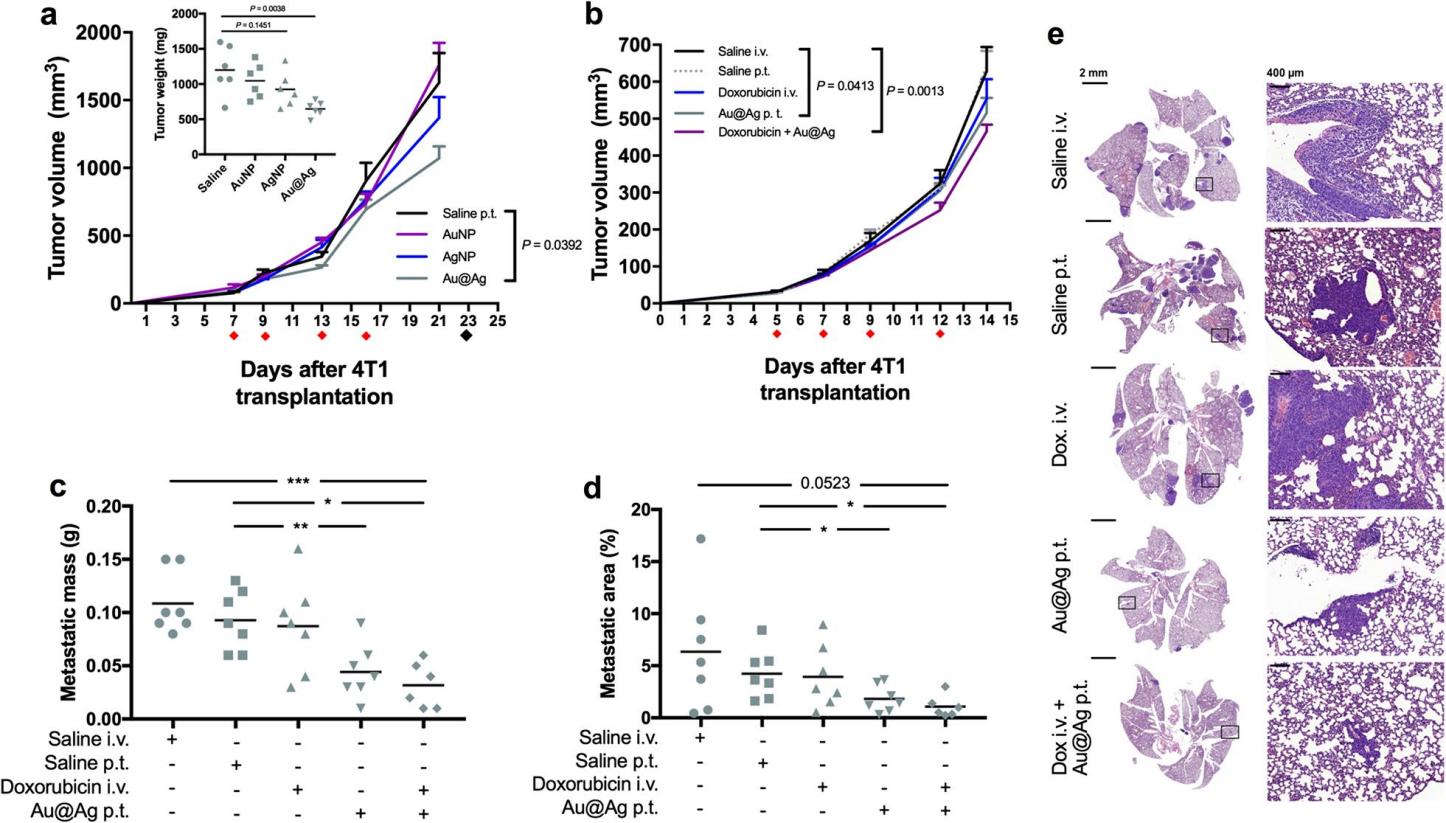
Au@Ag nanoparticles suppress metastasis in vivo. To test the in vivo activity of AgNP, AuNP and Au@Ag nanoparticles, 4T1 cells were transplanted into the mammary fat pad of Balb/c mice, then animals were divided into 4 groups (n = 6/group). Animals received nanoparticles peritumourally four times, and tumour sizes were repeatedly measured throughout the experimental period. Au@Ag nanoparticle treatments resulted in a significant suppression of 4T1 tumour growth (**a**). To investigate the anti-metastatic activities of Au@Ag nanoparticles, lower number of 4T1 cells were inoculated into the mammary fat pads of Balb/c mice then animals were divided into 5 groups (n = 6–7/group) to receive Au@Ag nanoparticles alone or in combination with doxorubicin. We found that Au@Ag alone and also in combination with doxorubicin hampered tumour progression significantly (**b**). After the last treatment (on day 12), animals were left untreated for further two weeks to let the metastatic lesions in the lungs grow to a potentially detectable level. On day 28 animals were euthanised and lungs were dissected. Au@Ag treatments alone and in combination with doxorubicin reduced the mass of metastatic tissue in the lung of the animals (**c**). Histopathology of the lung specimen also confirmed the anti-metastatic efficiency of Au@Ag, since the area of the metastatic lesions was significantly lower in nanoparticle-treated mice (**c**–**e**). *P ≤ 0.05; **P ≤ 0.01; ***P ≤ 0.001 indicates statistical significance (unpaired *t*-test)

Since AgNPs and Au@Ag nanoparticles suppressed the proliferation and invasion of 4T1 cells in vitro, we tested the in vivo performance of these particles as well, using a 4T1 cell-based tumour model. For this, 4T1 cells were transplanted into the thoracic mammary fat pads of Balb/c mice, and when the tumours became palpable animals were divided into 4 groups to receive peritumoural injections of saline, AuNP, AgNP and Au@Ag nanoparticles, respectively. Compared to saline obtaining animals, significant tumour growth inhibition was detected only in the Au@Ag receiving group at day 21 (P = 0.0392), which difference was strengthened when animals were dissected and tumour weights were measured at day 23 (Fig. 2a, Additional file 5). This result indicates that among the three tested metal nanoparticles types only Au@Ag exhibited moderate, however noteworthy anti-cancer activity in vivo.

Albeit we detected the above described anti-cancer effect of Au@Ag, unfortunately evaluation of possible metastasis suppressing activity of this nanoparticle was not possible under the applied conditions. Due to the high number of transplanted 4T1 cells the primary tumours grew fast and the animals had to be terminated according to animal handling rules and regulations before the metastatic tissue in the lungs could be developed reaching the threshold of statistical evaluation. As our main interest was to delineate the metastasis modulating ability of these nanomaterials, therefore, we performed a second in vivo experiment with modified conditions. Since among the previously tested nanoparticles only Au@Ag exhibited measurable anti-tumour activity, in this second in vivo experiment we employed Au@Ag nanoparticles either alone or together with intravenously administrated doxorubicin to test concomitantly how these hybrid nanoparticles would perform in combination with an anti-cancer drug already utilized in clinical practice. In line with these, tumour-bearing animals were treated with either Au@Ag alone, or in combination with doxorubicin, and with their respective administration controls (Fig. 2b). The desired moderate tumour progression rate was achieved, granting a notably longer experimental period for the development of measurable metastatic lesions. The animals received four treatments over 12 days, then were left untreated for further 2 weeks before they were euthanized. After the last treatments at day 12, significantly smaller tumour sizes were detected both in Au@Ag-exposed as well as in Au@Ag + doxorubicin receiving animals (Fig. 2c, Additional file 6a). More importantly, the metastatic mass in the lungs (Fig. 2c), the number of surface metastatic nodules (Additional file 7) and also the extent of the metastatic area (Fig. 2d,e, Additional file 6b) were significantly decreased in both Au@Ag nanoparticle receiving groups compared to the control animals. These findings indicate that Au@Ag nanoparticles attenuate metastasis.

Additionally, according to an obligatory protocol, a standard toxicology experiment was performed where Balb/c mice obtained intravenous Au@Ag injections four times, then essential toxicological parameters were determined. Au@Ag treatments did not influence either body weight or the weight of the animals’ liver or spleen, indicating no systemic toxicity of the applied treatments (Additional file 8).

### Metal nanoparticles suppress cancer-promoting activity of tumour-associated fibroblasts

We found that the remarkable in vivo metastasis-suppressing capability of Au@Ag nanoparticles was not coupled to a strong primary tumour growth inhibition, suggesting that nanoparticles affect notably the metastasis-promoting cells in the reactive stroma. Histopathology data on in vivo 4T1 tumours show recruitment of massive amounts of host-derived fibroblasts into the tumour microenvironment (data not shown). As cancer-associated fibroblasts represent the largest portion of the reactive tumour stroma, we decided to investigate further whether metal nanoparticle treatments influence the metastasis-promoting activity of the tumour microenvironment.

**Fig. 3:**
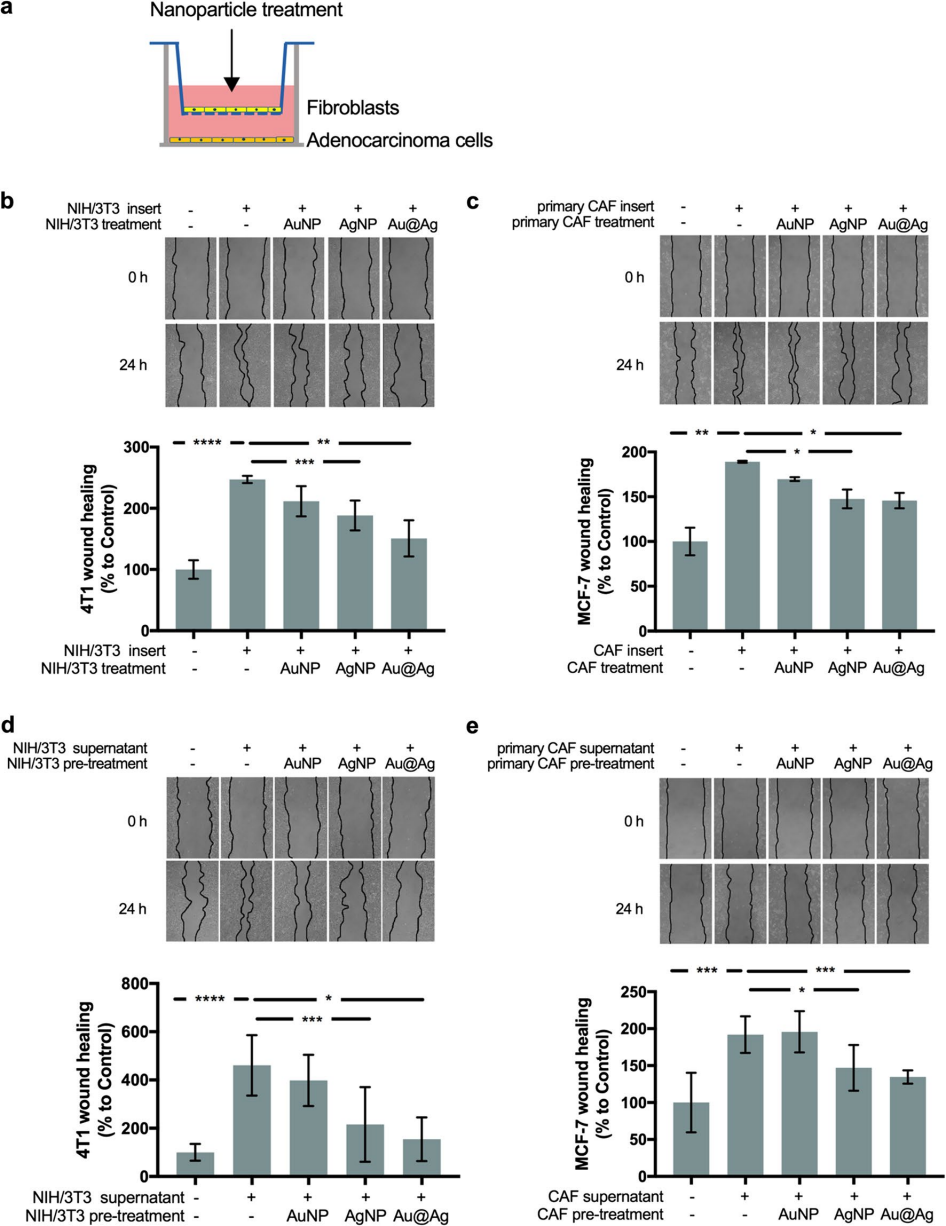
AgNP and Au@Ag treatments modulate the tumour-promoting activity of cancer-associated fibroblasts. To analyse whether nanoparticle treatments can affect the tumour-promoting activity of CAFs, first, a co-culture system was established in which 4T1 or MCF-7 tumour cells were grown in the lower chambers and NIH/3T3 or primary CAF cells were cultured separately in the upper compartments of 0.4 μm pore-sized transwell inserts (**a**). Wound healing activity of 4T1 or MCF-7 cells was monitored after selective treatment of NIH/3T3 or CAF fibroblast cells with non-toxic concentrations of metal nanoparticles. Experiments revealed that AgNPs and Au@Ag nanoparticles disrupted the fibroblast-induced, elevated wound healing activity of both 4T1 (**b**) and MCF-7 (**c**) cells. Similar experiments were performed using the conditioned media of AgNP, AuNP or Au@Ag nanoparticle-pre-treated NIH/3T3 and primary CAF cells. When adenocarcinoma cells were treated with the fibroblast-conditioned media, cell-free zones were scratched to the confluent cancer cell layers, then the area of the cell free zones were measured 24 h later to calculate wound healing activity. AgNP and Au@Ag treatments decreased the wound healing-promoting activity of both NIH/3T3 (**d**) and primary CAF cells (**e**). *P ≤ 0.05; ***P ≤ 0.001; ****P ≤ 0.0001 indicates statistical significance (unpaired *t*-test).

To this, we established in vitro co-culture systems using mouse NIH/3T3 fibroblasts and 4T1 adenocarcinoma cells and also, a similarly arranged human model system using patient-derived primary CAFs (characterised in Additional file 9) and MCF-7 human adenocarcinoma cells. In these co-culture systems, confluent fibroblast layers were cultured on the surface of 0.4 μm pore sized transwell inserts, while adenocarcinoma cells were grown in the lower chamber and their migration activity was monitored by wound healing assays (Fig. 3a). Fibroblasts and tumour cells were co-cultured for 24 h, then in order to selectively expose tumour-associated fibroblast cells nanoparticles were added to the upper chamber in non-toxic concentrations (20 μM silver or/and 25 μM gold, respectively). Concomitantly to the addition of the nanoparticles, wounds were scratched into the confluent layer of adenocarcinoma cells in the lower chamber, and 24 h later the sizes of the cell-free zones were evaluated to assess wound healing activities of 4T1 or MCF-7 cells, respectively (Fig. 3b, c).

In the presence of both NIH/3T3 and patient-derived cancer-associated fibroblast cells, tumour cell migration increased significantly, supporting that the attendant fibroblast cells promote the motility of adenocarcinoma cells. AgNP and Au@Ag treatments significantly suppressed the tumour cell-promoting activity of fibroblasts, which feature manifested in reduced wound closure, thus slower migration of adenocarcinoma cells. In contrast, no differences in cancer cell migration ability were observed when AuNPs were present in the co-cultures. At the end of each experiment, fibroblast containing inserts were stained with crystal violet and were inspected to ensure that confluent cell layers remained intact throughout the treatment, so nanoparticles could not diffuse through the membrane into the lower chamber (data not shown). Also, to exclude the possibility that nanoparticles provoke fibroblast membrane damage resulting ultimately in cell membrane leakage, NIH/3T3 fibroblasts were treated with AuNP, AgNP and Au@Ag nanoparticles (in 20 μM silver or/and 25 μM gold concentrations), and 24 h later lactate dehydrogenase (LDH) activity was measured in the supernatant (Additional file 10).

As the LDH activity was not elevated in the supernatant of nanoparticle treated fibroblasts compared to untreated cells, we assumed that the previously observed tumour cell proliferation-modulatory effects of fibroblasts must be the result of the altered secretory profiles and not of cell membrane-leaking factors. To further verify that nanoparticle treatments can modify the secretory profile of fibroblasts and thereby the cancer cell-supporting function of these stroma cells can be altered, NIH/3T3 and patient-derived CAF cells were exposed to nanoparticles, then nanoparticle containing media were replaced by serum-free media in order to collect fibroblast-secreted factors. The obtained conditioned media were concentrated, and applied on 4T1 or MCF-7 adenocarcinoma cells for 24 h, then cell migration was monitored by wound healing assay (Fig. 3d, e). Addition of untreated fibroblast-derived conditioned media to the adenocarcinoma cells increased cell migration significantly. A comparably elevated migration was observed when 4T1 or MCF-7 were exposed to media collected from AuNP-pre-treated fibroblasts. More importantly, when conditioned media of either AgNP- or Au@Ag nanoparticle-pre-treated NIH/3T3 or primary human fibroblasts were applied on cancer cells, the tumour cell-promoting effects of the fibroblast-derived media diminished, resulting in a comparable migration activity as non-stimulated counterparts. A similar experiment was performed using BrdU incorporation assay as a read-out to measure cell proliferation. The results obtained from the BrdU assays, validated all the previously observed attenuating effects of metal nanoparticles on the cancer cell migration-promoting effects of fibroblasts (Additional file 11).

**Fig. 4:**
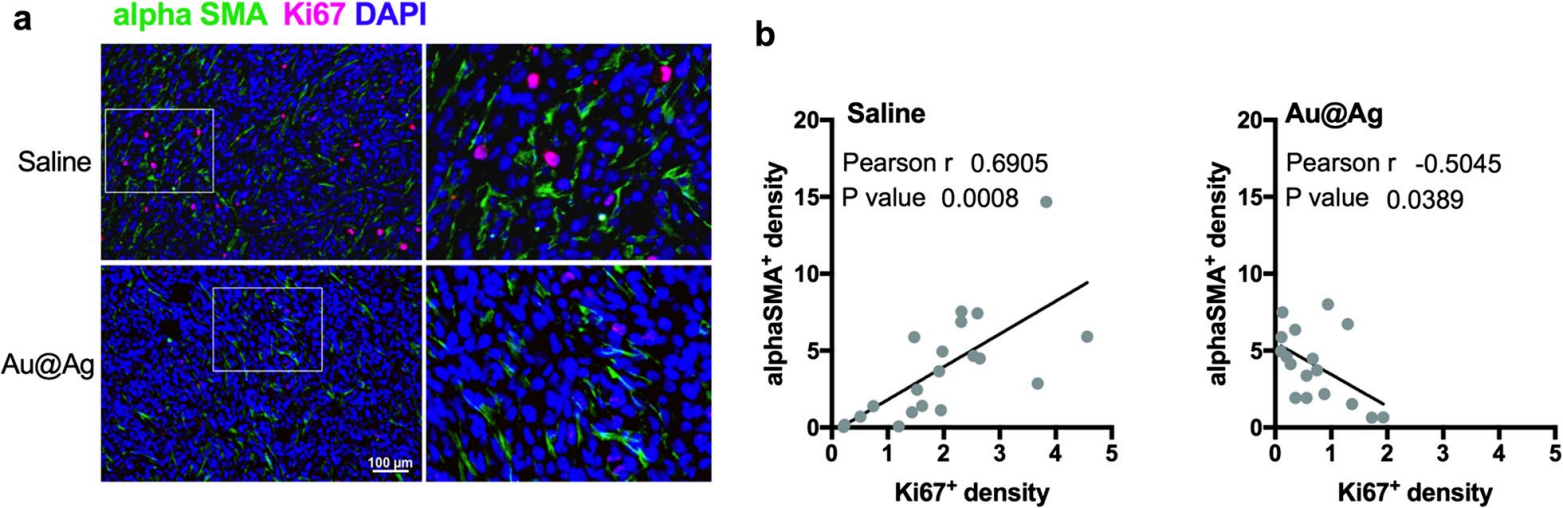
Au@Ag treatments decrease the number of proliferating cancer cells in fibroblast-rich tumour microdomains. High number of Ki67-positive cells can be identified in the alphaSMA-positive fibroblast-rich microdomains in saline-treated 4T1 tumours, while the number of proliferating tumour cells is markedly lower in the Au@Ag-treated tumours (**a**). In saline-treated tumours, a positive correlation can be identified between the numbers of Ki67- and alphaSMA-positive cells indicating that there is a noticeably higher proliferation activity in the fibroblast-rich regions of the cancerous tissue. On the other hand, loss of the positive correlation between Ki67- and alphaSMA-positive cells can be identified in Au@Ag-treated tumours, since the nanoparticle treatment decreased the number of Ki67-positive cells without influencing the number of infiltrated cancer-associated fibroblasts (**b**)

To demonstrate that these fibroblast-modulating effects of metal nanoparticles can be observed not only in cell culture, but also in vivo, we performed immunohistochemistry on the tumour samples obtained from mice treated with Au@Ag nanoparticles (tumours from animals in experiment shown on Fig. 2b–e). Tumour sections were counterstained with an antibody recognising the CAF marker alphaSMA-, as well as with proliferation marker Ki67-specific antibodies (Fig. 4a). Subcutaneous tumour regions were photographed and fibroblast-rich and fibroblast-poor fields were analysed. We observed higher number of Ki67-positive tumour cells within the fibroblast-rich regions, whereas cancer cells within tumour zones infiltrated with lower numbers of CAF cells were less proliferative (Additional file 12). Although this observation shows that CAF cells and proliferating tumour cells are embedded in the same niche of the tumour tissue, it also suggests that the presence of host-derived CAF cells promote the proliferation activity of the xenografted 4T1 cells. Most importantly, when tumours were treated with Au@Ag nanoparticles, a reduced number of Ki67-positive tumour cells were identified in the fibroblast-rich microdomains. To strengthen this latter observation, we analysed 4–5 photographs of each tumour sample in identical anatomical regions, and the relationship between Ki67- and alphaSMA-positive cells was tested by Pearson correlation test. A strong positive correlation between Ki67- and alphaSMA-positive cell density was found in saline-treated tumours (Fig. 4b). In the Au@Ag nanoparticle-treated tumours a lower Ki67-positive cell density was identified, whereas no change was observed in the number of alphaSMA-positive fibroblasts (Additional file 13). Hence, the nanoparticle-induced modulation of the tumour microenvironment resulted in a loss of positive correlation between cancer-associated fibroblasts and proliferative tumour cells. This observation suggests that Au@Ag nanoparticles suppress the cancer cell proliferation-promoting activity of CAF cells not only in vitro, but in vivo as well.

### AgNP and Au@Ag nanoparticles trigger expressional changes in metastasis-related genes

**Fig. 5:**
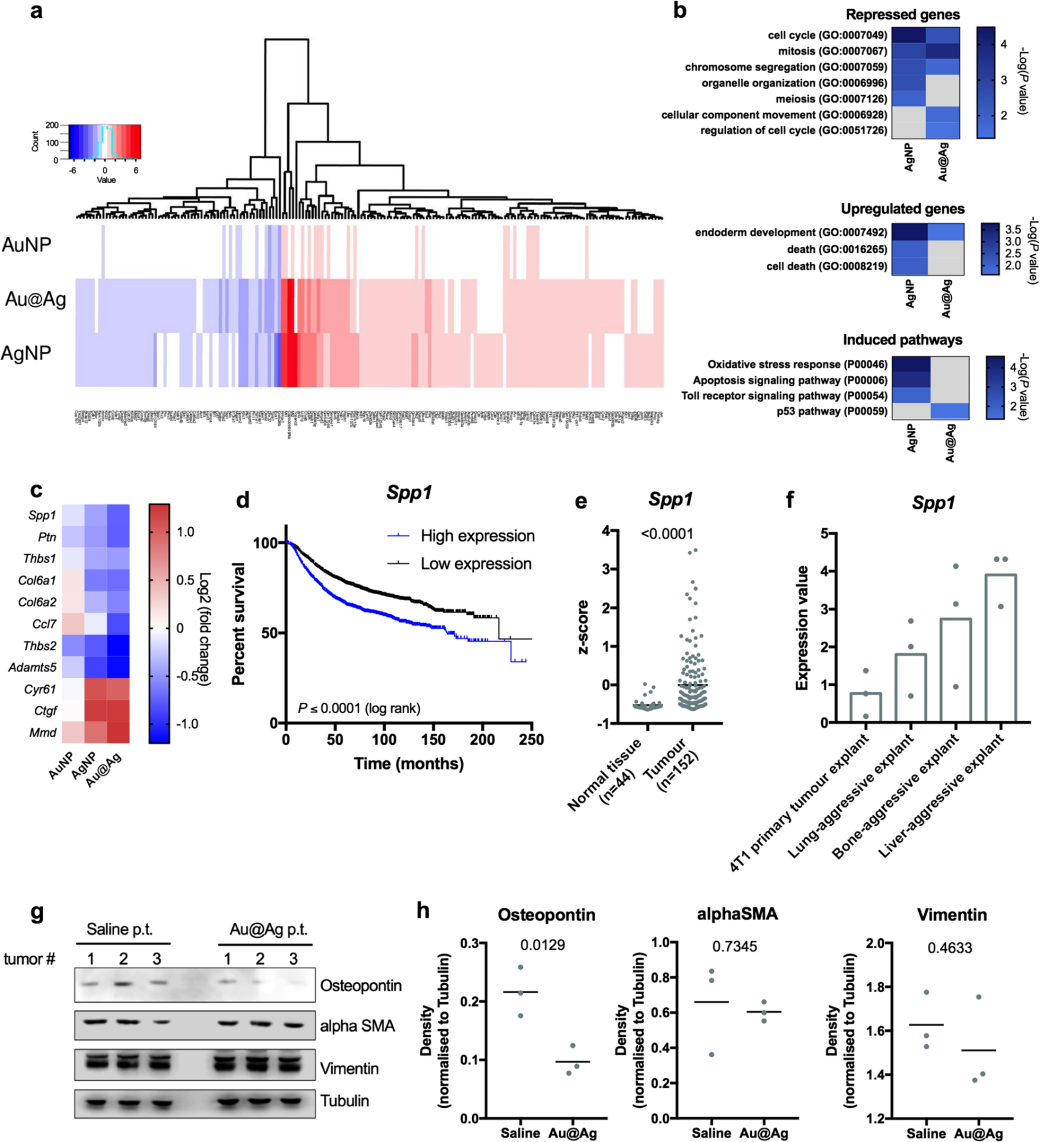
AgNP and Au@Ag treatments induce similar transcriptomic changes in CAFs. In order to reveal potential mechanisms in the background of the tumour modulatory effects of CAFs, NIH/3T3 fibroblasts—co-cultured with 4T1 tumour cells—were treated with nanoparticles then transcriptome analysis was performed. Heat map showing the observed gene expressional changes indicates that AgNP and Au@Ag nanoparticle treatments induced similar transcriptomic profiles, with significant transcriptomic alterations in several hundred genes (**a**). Gene ontology analysis of repressed and induced genes as well as of upregulated pathways was performed to link the observed transcriptomic changes to biological functions. Both AgNP and Au@Ag nanoparticles repressed the transcription of cell cycle- and cell division-related genes, however, only AgNPs induced cell death-associated gene expressional changes (**b**). Among the repressed and upregulated genes, several genes have secreted protein products which are related to cancer metastasis. AgNP and Au@Ag nanoparticle treatments decreased the majority of these metastasis-related genes in cancer associated fibroblasts (**c**). Kaplan–Meier plot of breast cancer patients with low and high *Spp1* expression indicates that the elevated expression of *Spp1* significantly worsens the survival of patients highlighting the clinical significance of intratumoural Osteopontin levels (**d**). TCGA patient data indicates that *Spp1* expression is upregulated in breast cancer (**e**). Elevated *Spp1* expression is associated to metastasis in 4T1 tumour model (**f**). Au@Ag treatments do not influence the expression levels of CAF markers alphaSMA and Vimentin, while significantly reduce intratumoural Osteopontin expression in vivo (**g**, **h**). Full-length blots are presented as Additional file 18

To reveal the possible mechanisms behind the altered behaviour of cancer-associated fibroblasts which were induced by metal nanoparticle exposures, we selectively treated NIH/3T3 fibroblast cells with AuNP, AgNP and Au@Ag nanoparticles in our co-culture model (as in Fig. 3a), then total RNA was isolated from the fibroblast cells and was used to perform RNA-seq. AuNPs did not trigger notable gene expressional changes, while AgNP and Au@Ag treatments induced significant alterations in the transcriptome of cancer-associated fibroblast cells. The gene expression changes produced by AgNP and Au@Ag nanoparticles seem largely similar (Fig. 5a, Additional files 14, 15). It is also noteworthy that both AgNP and Au@Ag provoked the highest fold changes in metallothionein (*Mt1*, *Mt2*) and heme oxygenase (*Hmox1*) expressions.

To identify specific differences between the transcriptomic profiles of AgNP- and Au@Ag-treated cells and to link the obtained expressional changes to biological functions, gene ontology analysis was performed with the list of up- and downregulated genes using Panther-GO slim Biological Processes statistical overrepresentation test and Panther Pathways analysis. Regarding the repressed genes, statistically significant enrichment was observed in cell cycle- and cell division-related Gene Ontology groups, implying that both AgNP and Au@Ag nanoparticle treatments triggered cell cycle arrest in fibroblasts (Fig. 5b). Interestingly, significant enrichment in cellular component movement GO group was observed solely in Au@Ag nanoparticle-treated cells. As of upregulated genes, AgNP treatments induced the expression of genes belonging to cell death-related ontology groups, while these GO groups did not show significant enrichment upon Au@Ag exposures. Both AgNP and Au@Ag nanoparticle treatments led to statistically significant enrichments in endoderm development gene ontology group. More importantly, according to our transcriptome analysis, AgNPs activated oxidative stress response, apoptosis, and toll receptor-related signalling pathways, while Au@Ag nanoparticle treatments induced the expression of genes related to the p53 pathway (Fig. 5b).

Among the significantly up- and downregulated genes, we identified 11 hits, where the gene products are secreted by fibroblasts and have already been linked to cancer invasion and tumour metastasis (Fig. 5c, Table 1). From our transcriptome analysis four genes—presenting the most remarkable expressional suppression upon nanoparticle treatments [*Spp1* (Osteopontin)*, Ptn* (Pleiotrophin)*, Thbs2* (Thrombospondin 2), *Adamts5* (ADAM metallopeptidase with thrombospondin type 1 motif 5)]—were selected to evaluate whether a correlation between the altered expression level of these genes and cancer patient survival can be identified. For this analysis clinical data of a 3951 breast cancer cohort was used. Kaplan Meier-plots generated for the chosen genes revealed that high expression of *Spp1* reduced significantly breast cancer patient survival, indicating the clinical relevance of intratumoural Osteopontin levels (Fig. 5d, Additional file 16). Using gene expression data available at TCGA database of patients diagnosed with breast cancer and of matching healthy samples, we found that among the identified 11 hits *Spp1* (Osteopontin) shows the highest tumour-specific upregulation (Fig. 5e, Additional file 17). Furthermore, analysis of reported expression data on metastatic sub-populations of 4T1 breast cancer revealed that elevated *Spp1* expression is associated to lung, bone and liver-specific metastatic activity as well (Fig. 5f). This evidence supports that (i) *Spp1* expression is upregulated in breast cancer, (ii) it is linked to metastasis and (iii) its high expression is coupled to poor patient survival. Thus, the molecular mechanisms modulating *Spp1* expression for example by nanoparticle administration are fundamentally intertwined with therapy outcome and life expectancy. Based on these findings we examined in vivo whether Au@Ag nanoparticle treatments would lead to decreased expression of *Spp1*. Using western blot analysis, we quantified the intratumoural levels of Osteopontin as well as of the fibroblast markers alphaSMA and Vimentin in the Saline- and Au@Ag-treated 4T1 tumour samples (Fig. 5g) (in tumours obtained from animals in experiment shown on Fig. 2b–e). Densitometrical analysis revealed that while Au@Ag treatments did not influence the intratumoural protein levels of the fibroblast markers alphaSMA and Vimentin, these nanoparticles significantly reduced Osteopontin expression in the metastatic 4T1 tumours (Fig. 5h).

**Table 1:** Functions of cancer-associated genes showing expressional changes in CAFs upon AgNP and Au@Ag exposures

Hit	Protein product	Role in invasion and metastasis	References
*Spp1*	Osteopontin	Promotes metastasis and invasion	[42–44]
*Ptn*	Pleiotrophin	High Ptn expression is correlated with metastasis, regulates cancer cell migration	[45]
*Thbs1*	Thrombospondin-1	Deregulation of Thbs1 promotes tumour angiogenesis and metastasis	[46, 47]
*Col6a1*	Collagen type VI alpha 1 chain	Col6a1 expression is associated with tumour size and metastasis	[48, 49]
*Col6a2*	Collagen type VI alpha 2 chain	High Col6a2 expression is correlated with poor cancer survival	[50]
*Ccl7*	chemokine (C-C-motif) ligand 7/monocyte-chemotactic protein 3	Tumour-associated fibroblast-driven CCL7 promotes metastasis	[51, 52]
*Thbs2*	Thrombospondin-2	Cancer cell-derived Thbs2 induces fibroblast activation. Thbs2 promotes bone metastasis in prostate cancer	[53, 54]
*Adamts5*	ADAM metallopeptidase with thrombospondin type 1 motif 5	Adamts5 overexpression regulates invasion of non-small cell lung cancer	[55, 56]
*Cyr61*	Cystein rich angiogenic inducer 61	Promotes tumour cell extravasation and metastasis	[57]
*Ctgf*	Connective tissue growth factor	Promotes osteosarcoma angiogenesis	[58]
*Mmd*	Monocyte to macrophage differentiation protein	Regulates tumour growth in non-small cell lung cancer	[59]

## Discussion

In the last decade, owing to the unique biological features, like antibacterial, antiviral and antifungal activities, metal nanoparticles, especially AgNPs have gained enormous attention. Since the anti-cancer effects of silver nanoparticles have been proposed, an even greater impetus was given to demonstrate the applicability of these nanomaterials in cancer treatment. However, the de facto application of AgNPs in oncotherapy is fraught with limitations, for example by some undesired effects to non-cancerous cells [28]. In contrast, gold nanoparticles are fairly biocompatible, therefore they hold considerable therapeutic potential for future clinical applications. In view of these established features i.e. the prominent anti-cancer effects of silver nanoparticles and the biocompatible nature of gold nanomaterials, we generated a hybrid nanoparticle system and examined its modulatory effects on cancer cells as well as on the tumour-promoting features of cancer-associated fibroblasts.

AgNPs are generally considered to be toxic to living organisms limiting their utilisation in cancer therapy. Using in vitro and in vivo approaches, we identified several favourable properties and actions of Au@Ag nanoparticles over AgNPs. For instance, IC_50_ values indicated that Au@Ag nanoparticles were less cytotoxic toward all of the examined adenocarcinoma and non-cancerous fibroblast cells compared to AgNP nanoparticles, albeit being similarly cancer cell-specific. Further advantages of Au@Ag nanoparticles were also apparent when migration-inhibitory and cell-invasion modulating activities of sub-cytotoxic doses of Au@Ag and AgNP nanoparticles were tested. In this respect the performance of the less-toxic Au@Ag nanoparticles was again comparable to those of the more-toxic AgNP counterparts. Concomitantly, in vivo toxicology experiment verified that intravenous administration of Au@Ag nanoparticles displayed no systemic toxicity in mice. Importantly, the size-surface ratio of the applied AgNPs were comparable to that of Au@Ag particles, hence showing different cytotoxicity. We believe that such a difference presumably results as a consequence of different Ag ion releasing dynamics. It is well-known that the reactive silver ions released from the nanoparticle surface are responsible for the majority of the biological effects of silver-based nanomaterials, therefore larger differences in this chemical property can undoubtedly influence the in vitro and in vivo toxicity of the tested nanoparticles. It has been reported that AgNPs are not able to dissolve completely but they can become passivated after releasing a given amount of ions, resulting in an inactive core, which cannot release reactive ions anymore [29]. These remnants can accumulate in the body and can therefore induce either short-term or long-term adverse effects. A possible explanation for the lower toxicity of Au@Ag might be the lack of accumulation of such ultra-small silver nanoparticle remnants, instead, a biocompatible non-toxic gold core remains behind after Au@Ag action. Nevertheless, to verify this hypothesis further investigation is required.

After demonstrating the rationality and the suitability of using core–shell structured Au@Ag nanoparticles, we wanted to test whether the presence of such nanoparticles in the tumour microenvironment could as well influence the metastatic potential of the primary tumours. Since the nanoparticles applied throughout the experiments were not optimized for a sustained plasma stability, therefore we bypassed the reticuloendothelial system by administrating the nanoparticles directly into the tumour microenvironment. Interestingly, administrated Au@Ag nanoparticles within the tumour tissue inhibited 4T1 tumour growth only mildly, but a striking suppression in the metastatic activity of the primary tumours could be observed in the nanoparticle-treated animals. This observation suggests that the outstanding anti-metastatic activity of the Au@Ag nanoparticles is not linked to a strong tumour growth inhibitory potential. The fact that the two features (metastasis suppression and tumor growth inhibition) do not change concomitantly, cannot be considered unusual. Indeed, using the same 4T1 tumour metastasis model, Avgustinova et al. [30] found that the lack of activation of cancer-associated fibroblasts by Wnt7a led to a loss of metastatic potential which was not coupled to a reduced in vivo tumour growth. Since we found the same phenotype on Au@Ag treated mice, we speculated that these nanoparticles, apart from the observed direct effects on cancer cells, could also modulate the behaviour of metastasis-promoting stromal fibroblasts. This hypothesis prompted us to install an in vitro co-culture system in which the indirect effects of the nanoparticle treatments could be monitored. In the established co-culture models, CAF cells clearly manifested tumour cell-promoting characteristics, which were effectively counter-modulated by AgNP and Au@Ag nanoparticles. As the nanoparticle treatments did not trigger fibroblast cell death or membrane leakage, we presumed that an alteration in the secretory profile of the cancer-promoting fibroblasts was triggered by the applied nanomaterials. This theory was further strengthened by treatments with conditioned media derived from nanoparticle-exposed fibroblasts, which provoked a similar effect on the adenocarcinoma cells as was previously seen by direct exposures of the co-cultures to nanoparticles.

When tumours of mice treated with Au@Ag were examined, reduced numbers of proliferative cancer cells were found in the fibroblast-rich microenvironment. This attenuated proliferation of cancer cells within the fibroblast-abundant tumour microdomains further supports the concept that Au@Ag nanoparticles affect cancer cells both directly as well as indirectly via modulation of stromal fibroblast cells. However, it should also be mentioned that Au@Ag nanoparticle treatments don’t seem to influence the capability of cancer cells to recruit CAF cells, as we found comparable levels of intratumoural fibroblasts in Saline- and Au@Ag-exposed animals by histopathological evaluation and by western blotting.

In our quest to identify potential cellular components targeted by nanoparticles in cancer-associated fibroblasts and to reveal mechanistic details on the effects of nanoparticle treatments on CAF cells, we performed transcriptome analysis. To our knowledge these are the first reported RNA-seq data sets on metal nanoparticle-treated cancer-associated fibroblasts. The analysis revealed that several secreted factors already linked to cancer metastasis showed repressed expression upon metal nanoparticle treatments. Growth factors (e.g. *Spp1*, *Ptn*), cytokines (*CCl7, Mmd*), extracellular components and extracellular matrix modifying factors (*Thbs1*,* Col6a1* and *Col6a2* and *Adamts5*) were identified in the transcriptomic background of the modified fibroblast behaviour in our co-cultures. These findings support the idea that alterations in the secretory profiles of nanoparticle-exposed cancer-associated fibroblasts are responsible for the observed modulatory effects of CAF cells. More importantly, these factors might also serve as therapeutic targets in future developments aiming the tumour stroma, and for this purpose, their precise function in tumour cell—fibroblast crosstalk should be investigated. Using metadata analysis of clinical cohorts, reported datasets and TCGA patient data extraction, we identified *Spp1* as a particularly promising target, since the high expression of this gene in breast cancer patients is coupled to poor patient survival and metastasis. Moreover, our data on 4T1 challenged mice also indicated that Au@Ag treatments decrease the intratumoural expression of this novel cancer-related metastasis-promoting therapeutic target in vivo.

The whole transcriptome profiles of metal nanoparticle-treated cancer-associated fibroblast cells also reflected that AgNPs and Au@Ag nanoparticles induced mostly expressional changes of genes related to cell cycle and cell division, suggesting that such nanoparticles exert an anti-proliferative effect in this cell type. On the other hand, AgNPs induced cell death- and apoptosis-associated transcriptomic alterations, whereas Au@Ag nanoparticles did not trigger the expression of such genes, which notion further corroborates the better cytocompatibility of the hybrid nanoparticles over AgNPs. This observation supports the hypothesis that while AgNPs can likely release Ag ions rapidly, the release of toxic ions from the gold core can potentially be slower or less effective, resulting in a sustained and prolonged anti-proliferative effect of Au@Ag treatments. It is also noteworthy, that nanoparticle treatments did not modify the expression of CAF and myofibroblast marker genes (e.g. alphaSMA, Vimentin) implying that unlike other nanomaterials (e.g. carbon nanotubes [31]), our metal nanoparticles do not influence fibroblast differentiation.

AuNPs (the same particles used as seeds for the core–shell nanoparticle synthesis) were systematically applied in parallel experiments throughout the entire study and their effects were constantly monitored. This was done with the purpose to estimate the individual contribution of the gold-core part to the biological effects exerted by Au@Ag nanoparticles and to analyse the behaviour of both in vivo and in vitro models to gold exposures. Similarly to AgNPs and Au@Ag nanoparticles, AuNPs were also readily taken up by both adenocarcinoma and fibroblast cells, revealed by TEM analysis (data not shown). Surprisingly, upon treatments with AuNPs, we did not observe significant toxicity or anti-tumoural actions which, however, can be explained by the biocompatible, biologically inert features of citrate-coated gold-materials. This finding is in a good accordance with several recent observations, where the authors reported the lack of AuNP toxicity toward various fibroblast cells [32, 33]. On the other hand, some publications presented results showing that gold nanoparticles are able to trigger anti-tumoural effects, can suppress metastasis and reverse epithelial-to-mesenchymal transition in melanoma [34]. Furthermore, Zhao et al. [35] reported recently, that in their study AuNPs could reduce intratumoural fibroblast density, and enhanced the efficacy of cisplatin-based chemotherapy. These controversial results demonstrate that key physico-chemical properties of AuNPs, such as size, surface charge and capping materials can dramatically influence the behavior and feasibility of metallic nanoparticles. In our transcriptome analysis we did not observe significant gene expressional differences between control and AuNP-exposed fibroblasts, suggesting a lack of notable response from CAFs to gold exposures. However, this latter observation could also be explained by the resolution of the performed RNA-seq, which allowed only the markedly expressed genes to be involved in the analysis, and therefore, it cannot be excluded that AuNPs induced some changes in genes with low basal expression.

Nevertheless, the intracellular presence of the gold-core of Au@Ag nanoparticles can yield ultimate benefits. Since, in accordance with the literature [36], and based on our own observations (data not shown) the gold-cores of Au@Ag nanoparticles possess strong radiosensitising effect in cancer cells, which feature can be excellently exploited in radiotherapy. As a result, we assume that while the dissociative silver-shell can be considered as the active anti-cancer component, after the silver shell completely vanishes due to silver ion release, the residual gold-core in the tumour microenvironment could serve to enhance the efficiency of radiotherapy. This 2-in-1 formulation renders such Au@Ag core–shell nanostructures particularly attractive platforms for the development of clinically applicable therapeutic agents.

Finally, as in the clinical practice chemotherapy is realised mainly in a combinational manner, we investigated the suitability and the performance of locally administrated Au@Ag nanoparticles in combination with intravenous doxorubicin treatments. We found that the in vivo metastasis-suppressing activity of Au@Ag was maintained upon combinational doxorubicin administrations, and simultaneously, the most significant reductions in tumour sizes were observed by doxorubicin + Au@Ag nanoparticle-treated animals. This is a highly relevant finding, as it demonstrates that the powerful anti-metastatic and anti-cancer effects of Au@Ag are not compromised when another antineoplastic drug is introduced, and it emphasises the excellence of Au@Ag nanoparticles as combinational partners in chemotherapy. Hence we believe that core–shell Au@Ag nanoparticles hold exceptional potential in advanced oncotherapy approaches as chemotherapy adjuvants.

## Conclusions

In summary, this study provides compelling data on the suitability and in vitro and in vivo efficacy of gold-core silver-shell type nanoparticles as potent anti-metastatic and anti-cancer agents. Besides their low toxicity, we also demonstrate that Au@Ag nanoparticles achieve the outstanding metastasis-suppressing activity by (i) acting directly on adenocarcinoma cells via inhibiting their proliferation, (ii) as well as indirectly by affecting cancer-associated fibroblasts through attenuating their cancer-promoting capabilities and modulating their secretory profiles. Au@Ag nanoparticles, applied alone or in combination with other chemotherapeutic agents, exert potent anticancer capability due to the dissociative silver-shell, whereas the residual gold-core could serve to amplify the effects of radiation therapy. Considering the moderate success rate of conventional chemotherapy and frequent cancer reoccurrence, utilisation of Au@Ag nanoparticles could provide numerous advantages in multimodal treatment approaches.

## Methods

### Nanoparticle synthesis and characterisation

All chemicals applied upon the synthesis of the nanomaterials were purchased from Sigma-Aldrich. Citrate-stabilised silver nanoparticles (AgNP) were synthesised by chemical reduction using sodium borohydride. First 0.2 g of sodium citrate was dissolved in 75 mL deionised water with vigorous stirring. The solution was heated to 70 °C and 2 mL of 1 m/V% silver nitrate (AgNO_3_), then 2 mL of 0.1 m/V% NaBH_4_ solution was added drop-wise into the mixture. The resulting yellowish-brown suspension was stirred on 70 °C for an hour. The final colloid samples were stored at 4 °C. Citrate-stabilised gold nanoparticles (AuNP) were synthesised by a similar process as AgNP nanoparticles except that tetrachloroauric acid solution was used as initial gold source.

The gold-core silver-shell nanoparticles were obtained using a method previously reported [37]. The formerly produced gold nanoparticle suspension was used as a seed solution and the silver-shell was established by multiple rounds of silver ion reduction. First, 60 μL of l-ascorbic acid solution (100 mM), 15 μL of AgNO_3_ (100 mM), and 75 μL of sodium hydroxide (NaOH, 100 mM) were added to a beaker containing 10 mL of the as-prepared AuNPs at room temperature and pH = 8.5. The reaction was slowly stirred for 30 min before 60 μL of l-ascorbic acid (100 mM), 15 μL of AgNO_3_ (100 mM), and 75 μL of NaOH (100 mM) were again added. After three cycles, the resulting particles were centrifuged at 1800 rpm for 20 min and re-dispersed in 10 mL of 15 mM aqueous solution of trisodium citrate, to remove any remaining interfering components.

Silver concentration of the as-prepared AgNP and Au@Ag nanoparticles was verified by atomic absorption spectroscopy. For this, nanoparticles were disrupted in a hydrogen-peroxide:sulfuric acid mixture using a CEM MARS 5 microwave digestion system. Ag concentration was determined in a Perkin Elmer Atomic absorption spectrometer using AgNO_3_ standards.

The morphological characteristics of AgNPs, AuNPs and Au@Ag nanoparticles were investigated by transmission electron microscopy (TEM) using a FEI Tecnai G^2^ 20 X-Twin microscope at an acceleration voltage of 200 kV. The particle size distribution of the samples was assessed by dynamic light scattering (DLS) analysis using a Zetasizer Nano Instrument (Malvern, Worcestershire, UK). The optical properties of nanoparticles were studied by spectral analysis, and the absorbance spectra of nanoparticles were recorded within the range from 300 to 800 nm using an Ocean Optics 355 DH-2000-BAL UV–VIS spectrophotometer and a 10-mm path length quartz cuvette.

### Cell culture, assays and in vitro treatment conditions

All cell lines were purchased from ATCC and cultured under standard conditions. Briefly, 4T1 and MCF-7 adenocarcinoma cells were maintained in RPMI supplemented with 10% FBS, 2 mM l-glutamine, 0.01% streptomycin and 0.005% ampicillin, while NIH/3T3 fibroblasts were cultured in 4.5 g/L glucose DMEM supplemented with 10% FBS, 2 mM l-glutamine, 0.01% streptomycin and 0.005% ampicillin, respectively. MRC-5 human fibroblasts were maintained in EMEM complemented with 10% FBS, 2 mM l-glutamine, 0.01% streptomycin and 0.005% ampicillin.

Human patient-derived CAF cells were isolated from colon adenocarcinoma specimen resected at the Department of Surgery, University of Szeged, Hungary. The study was performed in accordance with appropriate guidelines and regulations, and the experimental protocols were all approved by the Human Investigation Review Board and Ethics Committee of the Albert Szent-Györgyi Medical and Pharmaceutical Centre at the University of Szeged (Ref#37/2006). The patient gave informed consent. For isolating fibroblast cells, the specimens were digested then cultured in selection RPMI medium supplemented with 10% FBS. When fibroblast cells reached confluence, cells were trypsinised and transferred to 10% FBS containing DMEM medium supplemented with 1% amino acid solution. Primary fibroblasts used for the experiments were passaged no more than 4 times.

To determine IC_50_ values, 10,000 cells were seeded into the wells of 96 well plates, then cells were treated with nanoparticles in various concentrations on the following day for 24 h. Cell viabilities were assessed using standard MTT assay described before [38]. Viability values were expressed in the percentage of the untreated controls, then surviving curves and IC_50_ values were determined using a GraphPad Prism 7.0 software. Based on the IC_50_ values, cells were treated with non-toxic nanoparticle concentrations (e. g. AuNP—25 μM Au/0 μM Ag; AgNP—0 μM Au/20 μM Ag; Au@Ag—25 μM Au/20 μM Ag) for 24 h, upon further in vitro experiments.

To assess tumour cell migration, wound healing assays were performed. Cells were grown in 6 well plates until confluence, then each cell layer was scratched, creating crossing straight lines in the monolayer. Cell-free zones were photographed 0 and 24 h after scratching and wound healing activity was calculated using the following formula (cell free area t0 − cell free area treatment time/cell free area t0 of control − cell free area t0 of control treatment time) × 100. In case of fibroblast migration tests, instead of measuring the cell-free area, the number of individual migrating cells was determined. At the end of the wound healing assays, cells were collected and stained with Annexin V/PI by following the instructions of the suppliers (Life Technologies). Fluorescent intensities of 10,000 events were determined using a BD FACScalibur flow cytometer, and raw data was analysed by FlowJo V10 software. Wound healing and migration experiments were repeated at least three times using three biological replicates. Statistical analysis was performed using GraphPad Prism 7.0 software and representative experiments are shown.

For LDH activity measurements, NIH/3T3 cells were seeded into 96 well plates in 2000 cells/well density, then were treated with nanoparticles on the following day. After 24 h treatments, Cytotoxicity LDH Assay Kit-WST (Dojindo) was applied and the instructions of the suppliers were followed. Absorbance was read in a Synergy HTX multi-mode reader, and values were normalised to the positive control.

Transwell invasion assays were performed using the invasive 4T1 cell line. To do this, transwell inserts of 0.33 cm^2^ area and 8 µm pore size (Corning) were coated with 20 μg Engelbreth-Holm-Swarm sarcoma extracellular matrix gel (Sigma-Aldrich). 2.5 × 10^4^ cells were seeded in 1% FBS and nanoparticle containing media onto the surface of the coated transwells, then 20% FBS containing medium was applied in the lower chambers as a chemoattractant. After 24 h, samples were fixed, stained with crystal violet and transmigrated cells were counted using a phase-contrast microscope. Transwell invasion experiments were repeated at least three times using three biological replicates. For statistical evaluation GraphPad Prism 7.0 software was used and the results of one representative experiment are demonstrated.

### PKH26 labelling and flow cytometry

4T1 cells were fluorescently labelled using the PKH26 Red Fluorescent Linker Kit (Sigma-Aldrich). For this, 5 million 4T1 cells were pelleted and stained by following the instructions of the suppliers. Fluorescent 4T1 cells were mixed with non-labelled NIH/3T3 fibroblasts in 1:1 ratio, then 1.5 × 10^5^ cells were seeded into the wells of 6 well plates containing 1 cm diameter coverslips. Co-cultures were treated with nanoparticles on the following day, then after 24 h treatments, coverslips were collected, cells were fixed with 4% PFA, then samples were visualised under an Olympus BX51 fluorescent microscope. The remaining cells were trypsinised and fluorescent intensity of the sample was measured using BD FACScalibur flow cytometer. To identify labelled 4T1 cells and non-labelled NIH/3T3 cells, gating strategy was set according to dot plot profiles of fluorescently-labelled 4T1 monocultures.

### Animal studies

All the animal experimental protocols were approved and performed according guidelines and regulations of the Ethics Committee of the Biological Research Centre of the Hungarian Academy of Sciences and the Hungarian National Animal Experimentation and Ethics Board, in possession of an ethical clearance (number: XVI./1489/2014). 4T1-based tumour metastasis model has been applied as reported previously [39]. More precisely, for in vivo tumour growth, 100,000 4T1 cells were suspended in 100 µL RPMI and the cell suspensions were injected into the thoracic fat pad of 6–8 week old female Balb/c mice. When tumours became palpable, animals received nanoparticle treatments via peritumoural injections (V = 4 × 20 μL). Doses were the following: 4.8 μmol/kg Ag upon AgNP and Au@Ag and 6 μmol/kg Au upon AuNP and Au@Ag treatments. Doxorubicin (Sigma) was applied intravenously in 4 mg/kg dose. Saline was used as administration control upon both peritumoural and intravenous injections. At the end of the treatment periods, animals were euthanised to perform necropsy. Tumours and vital organs were removed and measured, and lungs were photographed under a stereomicroscope to quantify metastatic nodules. Lung metastasis was further quantified by calculating the weight of the metastatic mass [metastatic mass (mg) = lung weight (mg) of tumour bearing mice − lung weight (mg) of healthy mice (2000 mg)], and by histopathology, where metastatic lesions were expressed as the percentage of the whole section area.

A standard toxicology experiment was also performed, in order to assess the systemic toxicity of Au@Ag nanoparticles upon the treatment period. To do this, 6–8 week old female Balb/c mice were divided into 3 groups (n = 3/group), then animals received Au@Ag via intravenous injections four times in 6 μmol/kg dose.

### Co-culturing

For tumour cell—fibroblast co-cultures, NUNC Polycarbonate Cell Culture Inserts with 0.4 μm pore diameter and of 3.14 cm^2^ culture area were applied. 4T1 or MCF-7 cells were cultured in the lower chambers of the 6 well plates, while NIH/3T3 or primary CAF cells were seeded on the surface of transwell inserts using the same cell density. When fibroblast cells reached confluence, inserts were transferred into the wells of the 4T1 cell containing 6 well plates. Tumour cells and fibroblasts were co-cultured for 24 h, then the established cancer-associated fibroblast cells were treated with nanoparticles, while wound healing assay was performed on the 4T1 cells in the lower chamber. At the end of the experiments, NIH/3T3 and CAF cells were fixed, stained with crystal violet and visualized in a phase-contrast microscope, to ensure that the treatments did not induce the loss of cell–cell junctions between fibroblasts.

### Fibroblast conditioned media

To obtain fibroblast secreted factors, NIH/3T3 or primary CAF cells were treated for 24 h with nanoparticles, then nanoparticle containing media were replaced by fresh serum-free media. After 24 h incubation, conditioned media were collected, concentrated with Amicon Ultra 4 K centrifugal filter devices, then protein concentrations of the obtained solutions were measured with BioRad Protein Assay.

### BrdU incorporation assay

We measured the cell proliferation activity based on the S-phase-dependent incorporation of the nucleotide analogue 5-bromo-2′-deoxyuridine (BrdU) using Cell proliferation ELISA kit (Sigma-Aldrich). For this purpose, 2000 cells were seeded into the wells of 96 well plates, on the following day cells were treated with fibroblast conditioned media (containing fibroblast secreted proteins in 1 μg/mL concentration) for 24 h, then BrdU assay was performed following the instructions of the suppliers. Experiment was repeated three times using three biological replicates, and one representative experiment is shown.

### RNA sequencing

To investigate transcriptomic changes upon nanoparticle treatments in cancer-associated fibroblasts, co-culture experiments were performed. In the applied co-cultures, 4T1 cells were grown on the surface of 3.14 cm^2^ sized NUNC Polycarbonate Cell Culture Inserts, while NIH/3T3 cells were cultured below in the chambers of a 6 well plate. After 24 h of co-culturing, fibroblast cells were selectively treated with nanoparticles for 24 h, then RNA was purified from the fibroblast cells by Qiagen RNeasy mini kit. RNA concentrations were measured in a Qubit 2.0 fluorometer (Life Technologies), then RNA qualities were assessed in an Agilent 2100 Bioanalyzer by employing Agilent RNA 6000 nano Chip. Using 1 μg total RNA, non-strand specific sequencing library was prepared by Illumina TruSeq RNA sample Prep v2 kit, following the LS protocol. The size distribution of the generated library was assessed using DNA 1000 Chip in an Agilent 2100 Bioanalyzer, then the sequencing library was quantified by applying NEBNext Library Quant Kit (New England BioLabs) in a PikoReal real-time PCR system. Following quantification, 2 × 75 paired-end sequencing was performed on the samples using MiSeq Reagent kit V3-150 in an Illumina MiSeq NGS platform.

### Bioinformatics

Primary sequence analysis (base calling, demultiplexing, fastq file generation) was done by Run-Time Analysis (RTA v1.18.54) component of MiSeq Control Software (MSC v2.6.2.1). Fastq sequence files were quality trimmed and filtered to remove low quality and short sequences using Trimmomatic v0.33 in paired-end mode using parameters TRAILING:10 SLIDINGWINDOW:4:20 MINLEN:36. Paired sequence reads were aligned to GRCm38.p5 mouse reference genome (downloaded from https://www.gencodegenes.org) using TopHat 2.0. The resulting binary alignment (bam) files were sorted and deduplicated with SAMtools, then differential gene expression analysis was done with Cuffdiff using a gene annotation file (gencode.vM12.annotation.gtf). Expression heatmap was visualised in RStudio using gplots and heatmap.plus packages. Using the expression data of significantly up- and downregulated genes and the online PANTHER statistical overrepresentation test, we performed gene ontology and PANTHER pathway analysis (https://www.pantherdb.org). Upon the analysis PANTHER GO-Slim Biological process was applied. Bonferroni correction was applied upon P-value calculation. Significant enrichments were considered when P ≤ 0.01. GraphPad Prism 7 software was used to represent P values in heat maps.

Clinical relevance of selected genes was demonstrated by the published survival data of patients with low or high expression profile of the given gene using the online tool available at https://www.kmplot.com [40]. Gene expression data of 196 TCGA patients was extracted by Rstudio software using FirebrowseR package. Only those patient data were included into the analyses where the vital status was indicated as “dead”.

Microarray data of metastatic 4T1 tumour sub-populations published by Tabariès et al. were obtained from the NCBI Gene Expression Omnibus Dataset Browser (https://www.ncbi.nlm.nih.gov/sites/GDSbrowser?acc=GDS5666) [41]. Extracted gene expression and survival data was analysed using GraphPad Prism 7 software and P values were calculated with either log rank or unpaired t test.

### Immunohistochemistry

3 randomly selected, formalin-fixed tissues from Saline- and Au@Ag-treated groups were routinely embedded in paraffin, then 4 mm thick paraffin slices were cut. Samples were deparaffinised, then heat-mediated antigen retrieval was performed using citrate buffer at pH = 6. Samples were blocked with donkey serum (Millipore) and stained with alphaSMA-specific antibody (1:200) (abcam, ab21027) then Alexa488-labelled donkey-anti-goat secondary antibody (abcam, ab150129) was applied. After alphaSMA staining, samples were blocked again using normal goat serum, and Ki67-specific antibody (abcam, ab15580) was used in 1:50 dilution followed by Dylight549-labelled goat anti-rabbit secondary (abcam, ab96984). After immunoreactions, samples were counterstained with DAPI and analysed by an Olympus BX51 fluorescent microscope.

For correlation analysis, 4–5 photographs were taken from the subcutaneous regions of each tumour, then the percentage of alphaSMA- and Ki67-positive cells was quantified using CellProfiler 2.2.0 software. Statistical evaluation of the obtained data was performed using GraphPad Prism 7 software by calculating the Pearson r value.

### Western blotting

3–3 randomly selected tumours from Saline and Au@Ag-treated animals were sliced and homogenised in ice-cold RIPA buffer supplemented with protease inhibitor cocktail (Calbiochem) using glass potter homogeniser. Homogenisation was facilitated by ultrasound sonication, and following centrifugation protein concentration of the supernatants obtained from each sample was determined using the BCA method. 10 μg total protein/sample was resolved on 10% polyacrylamide-SDS gels, then transferred to nitrocellulose membranes (Amersham). For the detection of Osteopontin, membranes were blocked with 5% BSA-TBST while 5% milk-TBST was used for alphaSMA, Vimentin and Tubulin detections. Primary antibodies were applied overnight (Anti-Osteopontin (abcam, ab63856), Anti-alphaSMA (abcam, ab5694), Anti-Vimentin (abcam, ab8978), anti-Tubulin (Sigma, T9026), then membranes were incubated with HRP-conjugated secondary antibodies (DAKO). Membranes were developed using ECL reagent (Immobilon) and the chemiluminescent signal was detected by a LI-COR C-DiGit western blot scanner system. Uncropped western blots are presented in the Additional file 18.

## Supplementary information

**Additional file 1.**TEM, UV–Vis and DLS analysis of the obtained metal nanoparticles. TEM analysis of the as-prepared nanoparticles indicates that all the three nanoparticle preparations have quasi-spherical morphology (**a**). UV–Vis analysis shows characteristic absorbance peak of AuNP at 530 nm wavelength. AgNP and Au@Ag nanoparticles have absorption maximum around 400 nm due to their characteristic surface plasmon resonance (**b**). DLS measurements indicate that AuNPs have a mean hydrodynamic diameter around 9 nm, while AgNPs are 12 nm and Au@Ag nanoparticle have 11 nm average diameter. DLS measurements indicate enlargement in size upon Au@Ag synthesis indicating the successful shell formation on the surface of the applied Au core particles. As the characteristic AuNP peak disappears in the UV–Vis spectrum of Au@Ag nanoparticles, we concluded that the silver coverage on the core surface is complete (**c**). Size distribution of the nanoparticles determined by TEM image analyses. Mean values are indicated in nm unit (**d**).

**Additional file 2.** Surviving curves of AgNP and Au@Ag nanoparticle treated adenocarcinoma cells. Adenocarcinoma (4T1, MCF-7) and fibroblast (NIH/3T3, MRC-5) cells were seeded into 96 well plates, then were treated on the following day with various concentrations of AgNP and Au@Ag (**a**) or AuNP (**b**) nanoparticles. X-axis indicates the corresponding metal concentration of the medium upon nanoparticle treatments. MTT assay was performed 24 h after the addition of the nanoparticles and surviving curves were determined using GraphPad Prism 7.0 software. IC_50_ values were calculated and are indicated on the plots in M unit.

**Additional file 3.** Nanoparticle treatments do not influence the migration activity of fibroblast cells. NIH/3T3 and MRC-5 fibroblasts were cultured in 6 well plates until they reached confluency, then wounds were scratched and cells were treated with nanoparticles in the indicated metal concentrations. AgNP and AuNP nanoparticle concentrations were determined based on the silver and gold content of the medium upon Au@Ag nanoparticle treatments to selectively mimic the effects of the core and of the shell part of the Au@Ag nanoparticles. 24 h after treatments, cell free zones were photographed and numbers of migrating cells were determined. Nanoparticle treatments in the applied concentrations did not affect either NIH/3T3 or MRC-5 fibroblast migrations.

**Additional file 4.** The inhibition of 4T1 and MCF-7 wound healing activity upon AgNP and Au@Ag nanoparticle treatments is not coupled to cytotoxicity. To verify that the observed inhibition of wound healing activity is not coupled to cytotoxicity, cells were collected after the wound healing assays, stained with Annexin V/PI and flow cytometry was performed to define the ratio, of early-, late-apoptotic and necrotic cells. Neither nanoparticles induced considerable apoptosis induction. As a positive control, tumour cells were pre-treated for 24 h with the well-characterised apoptosis inducer small molecule M627 in 10 M concentration.

**Additional file 5.** Au@Ag nanoparticles suppress 4T1 tumour growth. Tumour progression curves of each animal involved in the experiment. Day 0 indicates the time of 4T1 tumour cell inoculation. Red rectangles indicate treatment times while black rectangles show termination time of the experiment.

**Additional file 6.** Au@Ag alone and in combination with doxorubicin nanoparticles suppress metastasis in vivo. (**a**) Tumour progression curves of 4T1 tumours in every single animal involved in the experiment. Day 0 indicates the inoculation of the cells. Red rectangles indicate treatment times while black rectangles point the termination time of the experiment. (**b**) Histopathology of the lungs of animals involved in the experiment and used for morphometric analysis.

**Additional file 7.** Number of surface metastatic nodules on the lungs of the animals involved in the second in vivo experiment. *P ≤ 0.05; **P ≤ 0.01 indicates statistical significance (unpaired *t*-test).

**Additional file 8.** Intravenously administrated Au@Ag nanoparticles are not toxic in mice. To test the toxic effects of Au@Ag nanoparticles, 6–8 week old female Balb/c mice were divided into 3 groups (n = 3), and left untreated, or received saline as an administration control or Au@Ag nanoparticles in four times (at day 1, day 5, day 9 and day 12) intravenously. At day 20, animals were sacrificed and necroscopy was performed. Nanoparticle treatments did not influence the most important toxicology parameters as no differences were observed in the body, liver and spleen weights of the experimental animals between treated and control groups.

**Additional file 9.** Characterisation of human primary CAF cells. Colon tumour samples were dissected and fibroblasts were isolated as described in Methods section. To validate that the isolated cells are fibroblasts, cultures were stained against CAF markers alphaSMA and Vimentin. Immunocytochemistry shows that the isolated primary cells are positive to both CAF markers, therefore are fibroblasts. IC_50_ values were also established on these cells after 24 h of AgNP and Au@Ag treatments. IC_50_values are shown on the graph and expressed in M units. The obtained citotoxity profiles of AgNP and Au@Ag nanoparticles are comparable to those we observed on NIH/3T3 and MRC-5 cell lines.

**Additional file 10.** LDH activity in the supernatant of nanoparticle treated NIH/3T3 cells. As none of the nanoparticle treatments triggered a measurable LDH activity in the supernatants, we concluded that these nanoparticles did not induce membrane damage upon the applied treatment conditions.

**Additional file 11.** Pre-treatment of fibroblast cells with AgNP or Au@Ag nanoparticles modifies their cancer cell proliferation-promoting activity. NIH/3T3 or primary CAF cells were treated with nanoparticles in non-toxic concentrations, then nanoparticle containing media was replaced 24 h later by serum free medium. Media were conditioned for 24 h, concentrated, and then applied on either 4T1 or MCF-7 adenocarcinoma cells. Exposures to supernatants of AgNP or Au@Ag-pre-treated fibroblasts led to diminished promotion of 4T1 and MCF-7 proliferations determined by BrdU incorporation tests. *P ≤ 0.05; **P ≤ 0.01; ***P ≤ 0.001; ****P ≤ 0.0001 indicates statistical significance (unpaired *t*-test).

**Additional file 12.** Proliferating tumour cells and cancer associated fibroblasts are found in the same microdomains of 4T1 tumours. Saline and Au@Ag treated tumour samples were PFA fixed, embedded to paraffin and immunohistochemistry was performed on deparaffinised sections using proliferation marker Ki67 and fibroblast marker alphaSMA specific antibodies. In the saline treated samples, high Ki67 density can be observed in the microenvironment of the fibroblast cells. In contrast with these, in the Au@Ag treated tumours, almost no proliferating cancer cells can be observed in the fibroblast-rich regions.

**Additional file 13.** Percentage of alphaSMA and Ki67 positive cells in Saline and Au@Ag treated tumours. Au@Ag treatments did not modify the number of alphaSMA positive fibroblasts, but reduced significantly the number of Ki67 positive cells. ***P ≤ 0.001 indicates statistical significance (Unpaired *t*-test).

**Additional file 14.** Similarities and differences between AgNP and Au@Ag nanoparticle induced gene expressional changes in NIH/3T3 cells co-cultured with 4T1 tumour cells. X-axis represent the expressional changes upon AgNP treatments, while Y-axis represents Au@Ag triggered expressional changes. Both nanoparticle treatments induced primarily oxidative stress-response related genes, such as the Heme oxygenase 1 enzyme (*Hmox1*), or the antioxidant Metallothioneins (*Mt1*, *Mt2*). Interestingly, both treatments decreased the expression of the centrosomal protein CCDC28B (*Ccdc28b*). Only AgNP nanoparticle treatments upregulated the expression of the Acetoacetly-CoA Synthetase enzyme (*Aacs*) and the Transcription Elongation Factor A N-Terminal And Central Domain Containing 2 gene (*Tceanc2*) which is involved in ketone body metabolism and adipose tissue development. Au@Ag nanoparticle treatments decreased the expression of the interferon-induced antiviral enzyme 2'-5'-oligoadenylate synthase-like protein 2 (*Oasl2*), and the MMP-2 substrate cytokine Chemokine (C-C motif) ligand 7 (*Ccl7*) which attracts macrophages during inflammation and metastasis.

**Additional file 15.** Additional Table Significant alterations in the transcriptome of cancer-associated fibroblast cells after AgNP, AuNP and Au@Ag treatments.

**Additional file 16.** Kaplan–Meier plots of low and high expression levels of *Ptn*, *Adamts5* and *Thbs2* genes in breast cancer patients.

**Additional file 17.** TCGA expression data of selected genes in normal and matching cancerous breast cancer tissues.

**Additional file 18.** Uncropped version of western blots presented in Fig. 5.

## Data Availability

The datasets used and/or analysed during the current study are available from the corresponding author on reasonable request.

## Abbreviations

AgNPs: silver nanoparticles
AuNPs: gold nanoparticles
Au@Ag: gold-core silver-shell nanoparticles
CAFs: cancer-associated fibroblasts
EPR: enhanced permeability and retention effect
TEM: transmission electron microscopy
UV–Vis: ultraviolet–visible light absorption
DLS: dynamic light scattering
M627: 12H-benzo[alpha]phenothiazine
LDH: lactate dehydrogenase
BrdU: 5-bromo-2′-deoxyuridine
MTT: 3-(4,5-dimethylthiazol-2-yl)-2,5-diphenyltetrazolium bromide
SMA: alpha smooth muscle actin
Mt1: metallothionein 1
Mt2: metallothionein 2
Hmox1: heme oxygenase 1
Spp1: osteopontin
Ptn: pleiotrophin
Thbs2: thrombospondin 2
Adamts5: ADAM metallopeptidase with thrombospondin type 1 motif 5
Wnt7a: wnt family member 7A
Col6a1: collagen type VI alpha 1 chain
Col6a2: collagen type VI alpha 2 chain
Ccl7: chemokine (C-C-motif) ligand 7/monocyte-chemotactic protein 3
Cyr61: cystein rich angiogenic inducer 61
Ctgf: connective tissue growth factor
Mmd: monocyte to macrophage differentiation protein
