# Supplementary figures and images for: Core–shell nanoparticles suppress metastasis and modify the tumour-supportive activity of cancer-associated fibroblasts
Source: J Nanobiotechnology. 2020 Jan 21;18:18. doi: 10.1186/s12951-020-0576-x (PMC6974972; doi:10.1186/s12951-020-0576-x)

**Additional File 1.**


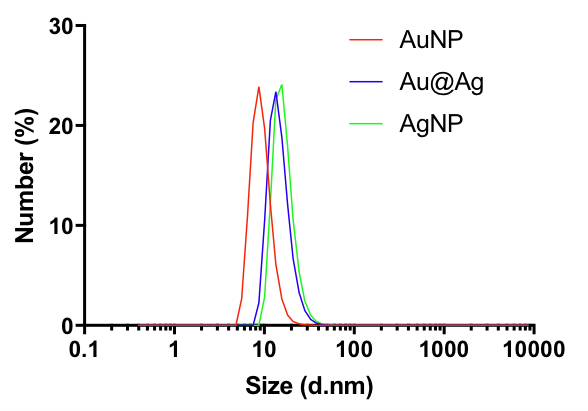


**a**

**b**

**c**


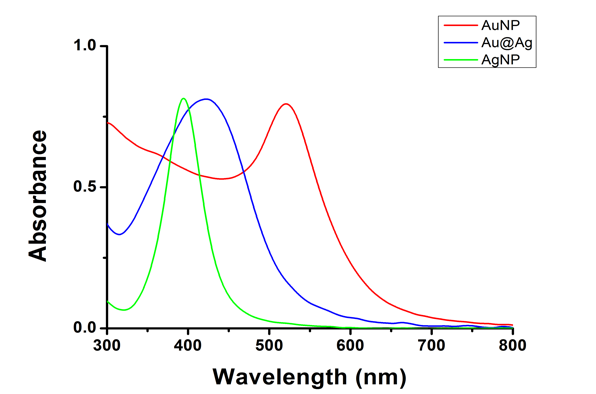

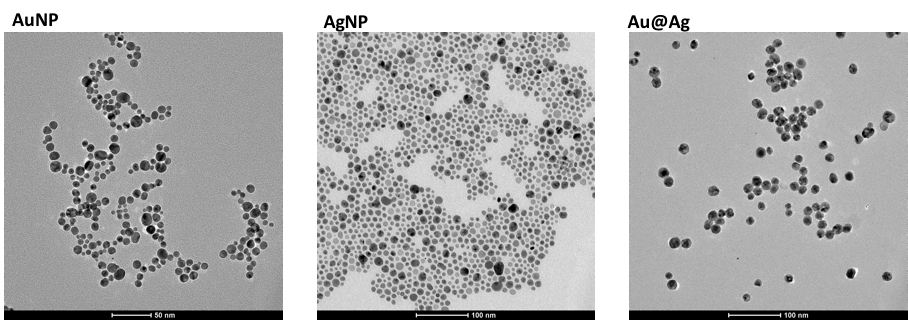


**d**

Supplement: Supplementary file 1 — Additional file 1.TEM, UV–Vis and DLS analysis of the obtained metal nanoparticles. TEM analysis of the as-prepared nanoparticles indicates that all the three nanoparticle preparations have quasi-spherical morphology (a). UV–Vis analysis shows characteristic absorbance peak of AuNP at 530 nm wavelength. AgNP and Au@Ag nanoparticles have absorption maximum around 400 nm due to their characteristic surface plasmon resonance (b). DLS measurements indicate that AuNPs have a mean hydrodynamic diameter around 9 nm, while AgNPs are 12 nm and Au@Ag nanoparticle have 11 nm average diameter. DLS measurements indicate enlargement in size upon Au@Ag synthesis indicating the successful shell formation on the surface of the applied Au core particles. As the characteristic AuNP peak disappears in the UV–Vis spectrum of Au@Ag nanoparticles, we concluded that the silver coverage on the core surface is complete (c). Size distribution of the nanoparticles determined by TEM image analyses. Mean values are indicated in nm unit (d). [file 12951_2020_576_MOESM1_ESM.docx]

**Additional File 2.**


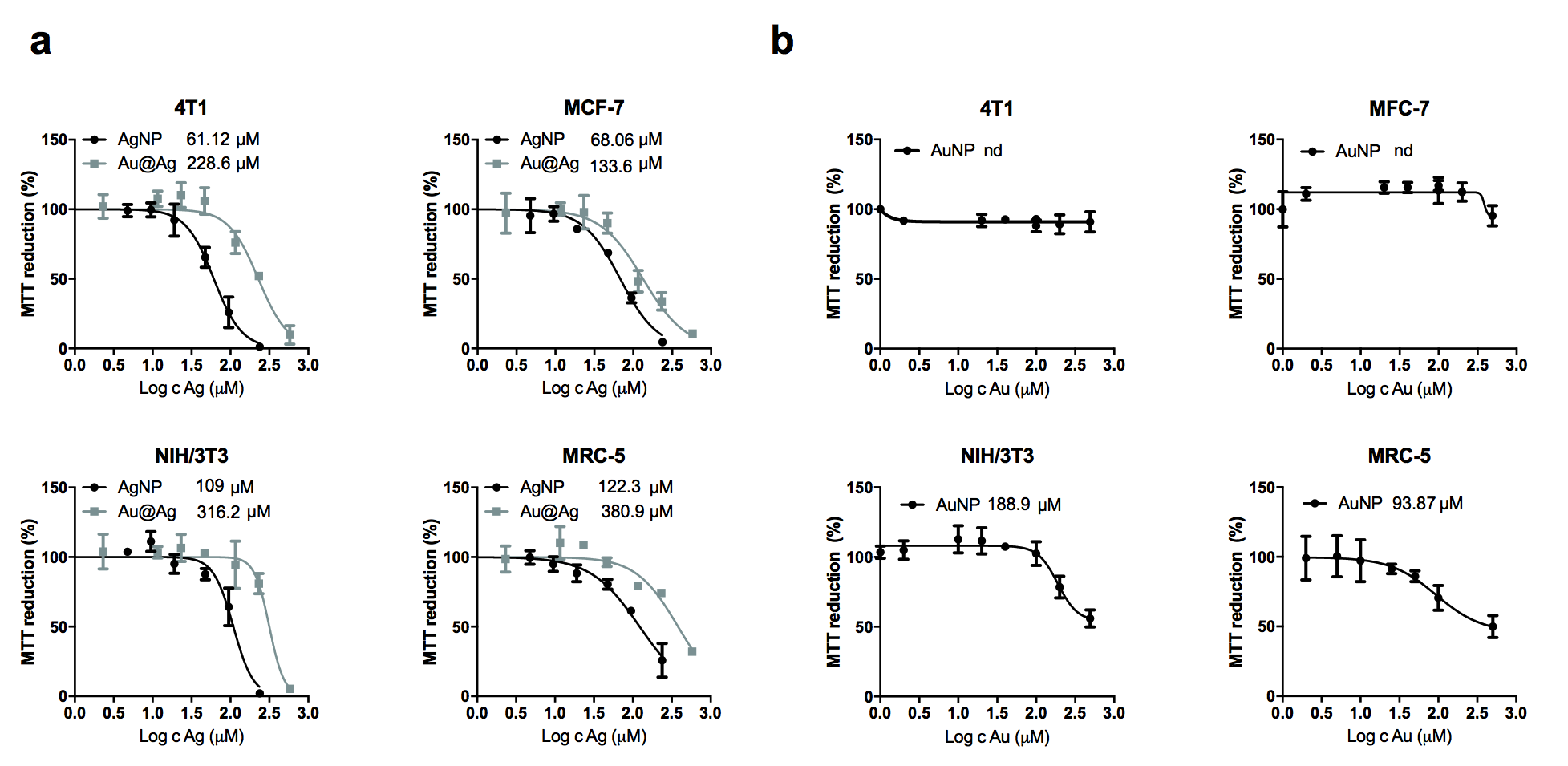

Supplement: Supplementary file 2 — Additional file 2. Surviving curves of AgNP and Au@Ag nanoparticle treated adenocarcinoma cells. Adenocarcinoma (4T1, MCF-7) and fibroblast (NIH/3T3, MRC-5) cells were seeded into 96 well plates, then were treated on the following day with various concentrations of AgNP and Au@Ag (a) or AuNP (b) nanoparticles. X-axis indicates the corresponding metal concentration of the medium upon nanoparticle treatments. MTT assay was performed 24 h after the addition of the nanoparticles and surviving curves were determined using GraphPad Prism 7.0 software. IC50 values were calculated and are indicated on the plots in M unit. [file 12951_2020_576_MOESM2_ESM.docx]

**Additional File 9.**


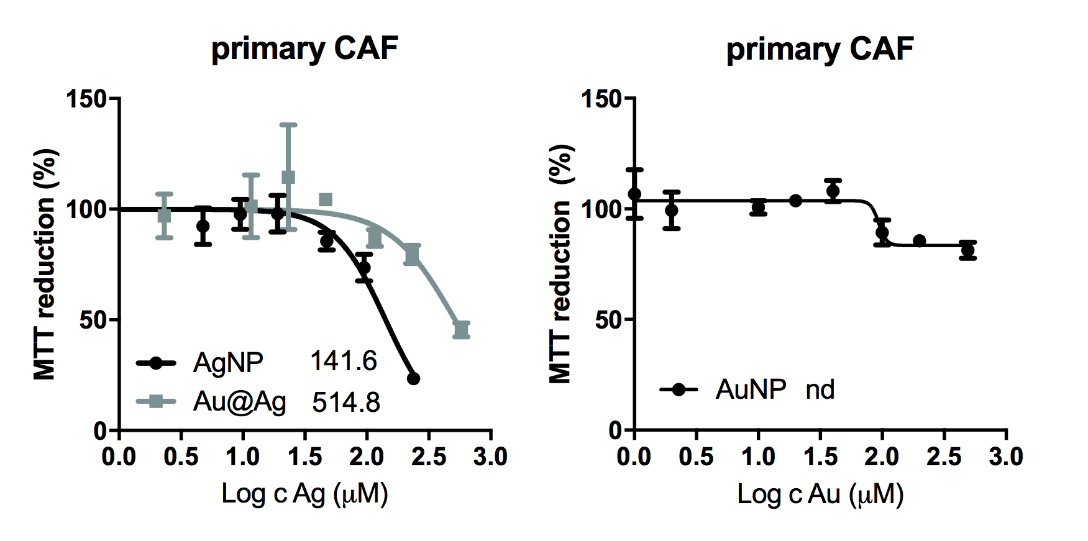

Supplement: Supplementary file 9 — Additional file 9. Characterisation of human primary CAF cells. Colon tumour samples were dissected and fibroblasts were isolated as described in Methods section. To validate that the isolated cells are fibroblasts, cultures were stained against CAF markers alphaSMA and Vimentin. Immunocytochemistry shows that the isolated primary cells are positive to both CAF markers, therefore are fibroblasts. IC50 values were also established on these cells after 24 h of AgNP and Au@Ag treatments. IC50values are shown on the graph and expressed in M units. The obtained citotoxity profiles of AgNP and Au@Ag nanoparticles are comparable to those we observed on NIH/3T3 and MRC-5 cell lines. [file 12951_2020_576_MOESM9_ESM.docx]

**Additional File 12.**


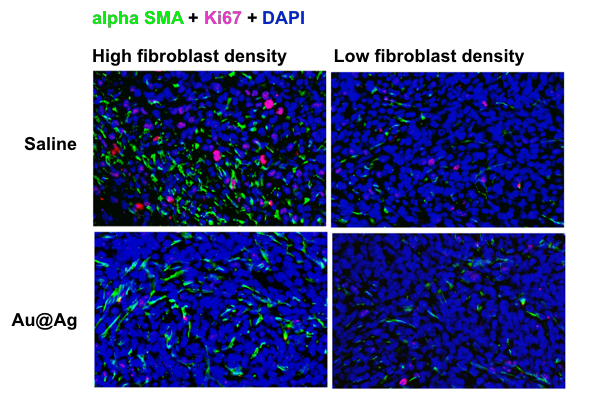

Supplement: Supplementary file 12 — Additional file 12. Proliferating tumour cells and cancer associated fibroblasts are found in the same microdomains of 4T1 tumours. Saline and Au@Ag treated tumour samples were PFA fixed, embedded to paraffin and immunohistochemistry was performed on deparaffinised sections using proliferation marker Ki67 and fibroblast marker alphaSMA specific antibodies. In the saline treated samples, high Ki67 density can be observed in the microenvironment of the fibroblast cells. In contrast with these, in the Au@Ag treated tumours, almost no proliferating cancer cells can be observed in the fibroblast-rich regions. [file 12951_2020_576_MOESM12_ESM.docx]

**Additional File 18.**

**
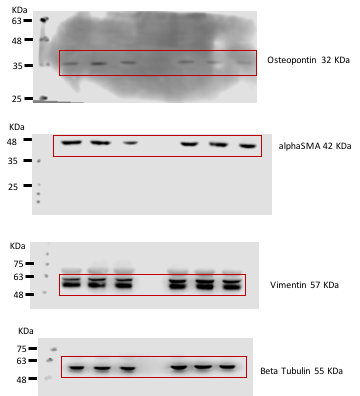
**

Supplement: Supplementary file 18 — Additional file 18. Uncropped version of western blots presented in Fig. 5. [file 12951_2020_576_MOESM18_ESM.docx]
