# Supplementary material for: Core–shell nanoparticles suppress metastasis and modify the tumour-supportive activity of cancer-associated fibroblasts
Source: J Nanobiotechnology. 2020 Jan 21;18:18. doi: 10.1186/s12951-020-0576-x (PMC6974972; doi:10.1186/s12951-020-0576-x)
Supplement: Supplementary file 4 — Additional file 4. The inhibition of 4T1 and MCF-7 wound healing activity upon AgNP and Au@Ag nanoparticle treatments is not coupled to cytotoxicity. To verify that the observed inhibition of wound healing activity is not coupled to cytotoxicity, cells were collected after the wound healing assays, stained with Annexin V/PI and flow cytometry was performed to define the ratio, of early-, late-apoptotic and necrotic cells. Neither nanoparticles induced considerable apoptosis induction. As a positive control, tumour cells were pre-treated for 24 h with the well-characterised apoptosis inducer small molecule M627 in 10 M concentration. [file 12951_2020_576_MOESM4_ESM.docx]

**Additional File 4.**
