# Supplementary material for: Core–shell nanoparticles suppress metastasis and modify the tumour-supportive activity of cancer-associated fibroblasts
Source: J Nanobiotechnology. 2020 Jan 21;18:18. doi: 10.1186/s12951-020-0576-x (PMC6974972; doi:10.1186/s12951-020-0576-x)
Supplement: Supplementary file 5 — Additional file 5. Au@Ag nanoparticles suppress 4T1 tumour growth. Tumour progression curves of each animal involved in the experiment. Day 0 indicates the time of 4T1 tumour cell inoculation. Red rectangles indicate treatment times while black rectangles show termination time of the experiment. [file 12951_2020_576_MOESM5_ESM.docx]

**Additional File 5.**
