# Supplementary material for: Core–shell nanoparticles suppress metastasis and modify the tumour-supportive activity of cancer-associated fibroblasts
Source: J Nanobiotechnology. 2020 Jan 21;18:18. doi: 10.1186/s12951-020-0576-x (PMC6974972; doi:10.1186/s12951-020-0576-x)
Supplement: Supplementary file 6 — Additional file 6. Au@Ag alone and in combination with doxorubicin nanoparticles suppress metastasis in vivo. (a) Tumour progression curves of 4T1 tumours in every single animal involved in the experiment. Day 0 indicates the inoculation of the cells. Red rectangles indicate treatment times while black rectangles point the termination time of the experiment. (b) Histopathology of the lungs of animals involved in the experiment and used for morphometric analysis. [file 12951_2020_576_MOESM6_ESM.docx]

**Additional File 6.**
