# Supplementary material for: Core–shell nanoparticles suppress metastasis and modify the tumour-supportive activity of cancer-associated fibroblasts
Source: J Nanobiotechnology. 2020 Jan 21;18:18. doi: 10.1186/s12951-020-0576-x (PMC6974972; doi:10.1186/s12951-020-0576-x)
Supplement: Supplementary file 7 — Additional file 7. Number of surface metastatic nodules on the lungs of the animals involved in the second in vivo experiment. *P ≤ 0.05; **P ≤ 0.01 indicates statistical significance (unpaired t-test). [file 12951_2020_576_MOESM7_ESM.docx]

**Additional File 7.**
