# Supplementary material for: Core–shell nanoparticles suppress metastasis and modify the tumour-supportive activity of cancer-associated fibroblasts
Source: J Nanobiotechnology. 2020 Jan 21;18:18. doi: 10.1186/s12951-020-0576-x (PMC6974972; doi:10.1186/s12951-020-0576-x)
Supplement: Supplementary file 8 — Additional file 8. Intravenously administrated Au@Ag nanoparticles are not toxic in mice. To test the toxic effects of Au@Ag nanoparticles, 6–8 week old female Balb/c mice were divided into 3 groups (n = 3), and left untreated, or received saline as an administration control or Au@Ag nanoparticles in four times (at day 1, day 5, day 9 and day 12) intravenously. At day 20, animals were sacrificed and necroscopy was performed. Nanoparticle treatments did not influence the most important toxicology parameters as no differences were observed in the body, liver and spleen weights of the experimental animals between treated and control groups. [file 12951_2020_576_MOESM8_ESM.docx]

**Additional File 8.**
