# Supplementary material for: Core–shell nanoparticles suppress metastasis and modify the tumour-supportive activity of cancer-associated fibroblasts
Source: J Nanobiotechnology. 2020 Jan 21;18:18. doi: 10.1186/s12951-020-0576-x (PMC6974972; doi:10.1186/s12951-020-0576-x)
Supplement: Supplementary file 10 — Additional file 10. LDH activity in the supernatant of nanoparticle treated NIH/3T3 cells. As none of the nanoparticle treatments triggered a measurable LDH activity in the supernatants, we concluded that these nanoparticles did not induce membrane damage upon the applied treatment conditions. [file 12951_2020_576_MOESM10_ESM.docx]

**Additional File 10.**
