# Supplementary material for: Core–shell nanoparticles suppress metastasis and modify the tumour-supportive activity of cancer-associated fibroblasts
Source: J Nanobiotechnology. 2020 Jan 21;18:18. doi: 10.1186/s12951-020-0576-x (PMC6974972; doi:10.1186/s12951-020-0576-x)
Supplement: Supplementary file 11 — Additional file 11. Pre-treatment of fibroblast cells with AgNP or Au@Ag nanoparticles modifies their cancer cell proliferation-promoting activity. NIH/3T3 or primary CAF cells were treated with nanoparticles in non-toxic concentrations, then nanoparticle containing media was replaced 24 h later by serum free medium. Media were conditioned for 24 h, concentrated, and then applied on either 4T1 or MCF-7 adenocarcinoma cells. Exposures to supernatants of AgNP or Au@Ag-pre-treated fibroblasts led to diminished promotion of 4T1 and MCF-7 proliferations determined by BrdU incorporation tests. *P ≤ 0.05; **P ≤ 0.01; ***P ≤ 0.001; ****P ≤ 0.0001 indicates statistical significance (unpaired t-test). [file 12951_2020_576_MOESM11_ESM.docx]

**Additional File 11.**
