# Supplementary material for: Core–shell nanoparticles suppress metastasis and modify the tumour-supportive activity of cancer-associated fibroblasts
Source: J Nanobiotechnology. 2020 Jan 21;18:18. doi: 10.1186/s12951-020-0576-x (PMC6974972; doi:10.1186/s12951-020-0576-x)
Supplement: Supplementary file 13 — Additional file 13. Percentage of alphaSMA and Ki67 positive cells in Saline and Au@Ag treated tumours. Au@Ag treatments did not modify the number of alphaSMA positive fibroblasts, but reduced significantly the number of Ki67 positive cells. ***P ≤ 0.001 indicates statistical significance (Unpaired t-test). [file 12951_2020_576_MOESM13_ESM.docx]

**Additional File 13.**
