# Supplementary material for: Core–shell nanoparticles suppress metastasis and modify the tumour-supportive activity of cancer-associated fibroblasts
Source: J Nanobiotechnology. 2020 Jan 21;18:18. doi: 10.1186/s12951-020-0576-x (PMC6974972; doi:10.1186/s12951-020-0576-x)
Supplement: Supplementary file 14 — Additional file 14. Similarities and differences between AgNP and Au@Ag nanoparticle induced gene expressional changes in NIH/3T3 cells co-cultured with 4T1 tumour cells. X-axis represent the expressional changes upon AgNP treatments, while Y-axis represents Au@Ag triggered expressional changes. Both nanoparticle treatments induced primarily oxidative stress-response related genes, such as the Heme oxygenase 1 enzyme (Hmox1), or the antioxidant Metallothioneins (Mt1, Mt2). Interestingly, both treatments decreased the expression of the centrosomal protein CCDC28B (Ccdc28b). Only AgNP nanoparticle treatments upregulated the expression of the Acetoacetly-CoA Synthetase enzyme (Aacs) and the Transcription Elongation Factor A N-Terminal And Central Domain Containing 2 gene (Tceanc2) which is involved in ketone body metabolism and adipose tissue development. Au@Ag nanoparticle treatments decreased the expression of the interferon-induced antiviral enzyme 2'-5'-oligoadenylate synthase-like protein 2 (Oasl2), and the MMP-2 substrate cytokine Chemokine (C-C motif) ligand 7 (Ccl7) which attracts macrophages during inflammation and metastasis. [file 12951_2020_576_MOESM14_ESM.docx]

**Additional File 14.**
