# Supplementary material for: Core–shell nanoparticles suppress metastasis and modify the tumour-supportive activity of cancer-associated fibroblasts
Source: J Nanobiotechnology. 2020 Jan 21;18:18. doi: 10.1186/s12951-020-0576-x (PMC6974972; doi:10.1186/s12951-020-0576-x)
Supplement: Supplementary file 15 — Additional file 15. Additional Table Significant alterations in the transcriptome of cancer-associated fibroblast cells after AgNP, AuNP and Au@Ag treatments. [file 12951_2020_576_MOESM15_ESM.docx]

**Additional File 15. Table**

| **GENE NAME** | **AuNP** | **AgNP** | **Au@Ag** |
| --- | --- | --- | --- |
| Col6a1 | 0.175186 | -0.624876 | -0.69215 |
| Ube2c | 0.219846 | -0.930071 | -0.629668 |
| Ifrd1 | -0.384578 | 1.12122 | 0.852763 |
| Psmc3 | 0.398072 | 0.382348 | 0.576666 |
| Tpra1 | -1.38343 | -0.966138 | -1.50365 |
| Kif20a | -0.0259723 | -1.09163 | -0.918556 |
| Calr | 0.0904371 | 0.541077 | 0.409244 |
| Nqo1 | 0.318987 | 1.81928 | 1.84511 |
| Mmd | 0.354565 | 0.892453 | 1.28963 |
| Atn1 | 0.122549 | -0.699437 | -0.17714 |
| Cnn2 | 0.229893 | 0.50522 | 0.603572 |
| Ndrg1 | 0.909686 | 1.63879 | 2.65105 |
| Hmox1 | 0.493074 | 4.55672 | 3.84637 |
| Ddx39 | 0.1193 | 0.425625 | 0.64981 |
| Dnajb1 | -0.012215 | 2.8769 | 1.97303 |
| Ampd3 | 0.734545 | 1.43385 | 1.80141 |
| Cdc20 | 0.00101724 | -1.18508 | -0.954748 |
| Ubc | 0.648055 | 1.94821 | 1.61227 |
| Gadd45b | 0.205731 | 0.35506 | 0.566859 |
| Hspa8 | -0.100151 | 0.82598 | 0.417333 |
| Sqstm1 | 0.421102 | 1.17172 | 1.05992 |
| Il13ra1 | -0.38441 | -0.543895 | -0.849581 |
| Serinc3 | 0.0531432 | 0.5995 | 0.384024 |
| Mafk | 0.114761 | 1.19062 | 1.20248 |
| Rars | 0.091479 | 0.696038 | 0.757426 |
| Map2k3 | 0.149499 | 0.703458 | 0.420777 |
| Ubb | 0.0671208 | 0.912024 | 0.672133 |
| Cdk1 | -0.337406 | -0.819197 | -0.582394 |
| Ctgf | 0.03 | 1.22307 | 1.23335 |
| Nudt4 | -0.190858 | -0.380408 | -0.479403 |
| Col6a2 | 0.167978 | -0.361602 | -0.574647 |
| Hmmr | -0.61367 | -1.07062 | -0.713267 |
| Mgat1 | -0.836517 | -0.425515 | -1.13945 |
| Prr11 | -0.2538 | -1.24554 | -0.98332 |
| Top2a | -0.272343 | -0.708935 | -0.670209 |
| Hsp90aa1 | -0.236975 | 1.35363 | 0.933295 |
| Klc1 | 0.204897 | 0.762116 | 0.171882 |
| Gadd45g | 0.803486 | 2.78196 | 2.02941 |
| Plk2 | 0.113135 | 0.90336 | 0.747355 |
| Esd | 0.0969411 | 1.37314 | 1.53641 |
| Tsc22d1 | -0.240093 | -0.878272 | -0.678695 |
| Dnajc3 | -0.122624 | 0.51327 | 0.65838 |
| Mtdh | -0.0335132 | 0.619296 | 0.223912 |
| Mtbp | 0.458555 | 1.00134 | 0.552173 |
| Csnk1e | 0.727731 | 0.668276 | 0.71877 |
| Zbtb11 | -0.30378 | 0.968207 | 0.532854 |
| Nde1 | -0.242553 | -0.682465 | -0.813398 |
| Adamts5 | -0.24606 | -0.897415 | -1.17259 |
| Abcc1 | 0.493359 | 0.836383 | 0.694469 |
| Thbs2 | -0.54829 | -0.783446 | -1.19911 |
| Tnfrsf12a | 0.0606698 | 0.637158 | 0.728006 |
| Cyp1b1 | -0.292496 | -0.914226 | -0.664714 |
| Dusp1 | 0.209119 | 1.08046 | 0.958652 |
| Tapbp | -0.446944 | -0.718133 | -1.57033 |
| Nedd4l | 0.247336 | 0.816749 | 0.748827 |
| Fth1 | 0.115807 | 0.981091 | 0.840252 |
| Ankrd1 | -2.90585e-05 | 0.718659 | 0.574007 |
| Fosl1 | 0.0968277 | 1.08841 | 0.936538 |
| Ehbp1l1 | -0.126636 | 0.744588 | 0.43797 |
| Stip1 | 0.204356 | 0.510424 | 0.520966 |
| Gsto1 | 0.216683 | 0.328324 | 0.573082 |
| Tollip | 0.145144 | 0.893317 | 0.529895 |
| Flnb | 0.121318 | 0.358432 | 0.510997 |
| Sat1 | 0.307541 | 0.779718 | 0.253529 |
| Esyt1 | 0.240881 | 0.751944 | 0.83689 |
| Ddit3 | 0.213804 | 0.758106 | 0.399061 |
| Taldo1 | -0.0690017 | 1.14583 | 1.08579 |
| Eya1 | -1.06693 | -2.37611 | -1.22862 |
| Mrpl30 | 0.535951 | 0.543995 | 0.926188 |
| Ubxn4 | 0.495826 | 1.25554 | 1.16346 |
| Csrp1 | 0.076607 | 0.613843 | 0.42129 |
| Rgs16 | -0.236254 | 0.967389 | 0.933296 |
| Prrx1 | -0.238574 | -0.301524 | -0.741151 |
| Atf3 | 0.0510678 | 3.5925 | 2.50722 |
| Fam107b | 0.380022 | 1.83 | 1.18403 |
| Atf6 | -0.12121 | 0.593201 | 0.432667 |
| Prdx6 | 0.093362 | 0.982216 | 0.845968 |
| Hspa5 | 0.00728768 | 0.938105 | 0.677355 |
| Nusap1 | -0.0745415 | -1.17321 | -1.73261 |
| Aurka | -0.0457821 | -0.627823 | -0.958241 |
| Procr | 0.446887 | 0.590729 | 1.1065 |
| Eif6 | 0.279839 | 0.509652 | 0.716931 |
| Tbl1xr1 | -0.604944 | -1.73314 | -1.10285 |
| Ccna2 | -0.0875602 | -0.948731 | -0.753756 |
| Ptx3 | -0.19987 | -0.418385 | -0.675799 |
| Gpsm2 | -0.472919 | -1.9527 | -1.56421 |
| Lce1g | 0.562542 | 2.82893 | 3.16112 |
| Dnajb4 | 0.286185 | 1.74311 | 1.55871 |
| Gclm | 0.569164 | 2.32706 | 2.38598 |
| Pip5k1a | 0.361746 | 1.00513 | 0.562828 |
| Cth | -0.223616 | 0.488343 | 0.377896 |
| Cyr61 | -0.0363206 | 1.09816 | 0.988912 |
| Tceanc2 | 0.153483 | 5.77223 | -0.338972 |
| Cap1 | 0.335728 | 0.642629 | 0.441102 |
| Plk3 | 0.505624 | 1.44005 | 1.17886 |
| Ccdc28b | -1.87689 | -3.66619 | -2.0844 |
| Pgd | 0.0589539 | 1.21569 | 1.14102 |
| Ccnl2 | -0.297885 | -0.726844 | -0.882779 |
| Cenpa | 0.286932 | -0.533009 | -0.96369 |
| Spp1 | -0.151754 | -0.438264 | -0.749905 |
| Dhx37 | -1.86034 | -1.38603 | -1.97537 |
| Aacs | 0.353717 | 2.21702 | 0.144868 |
| Oasl2 | -0.52828 | -0.79415 | -1.96688 |
| Hsph1 | 0.0207677 | 2.14908 | 1.40126 |
| Ptn | -0.28085 | -0.481492 | -0.753649 |
| Cnbp | -0.0612304 | 0.575672 | 0.440393 |
| Tspan9 | 0.718752 | 0.793044 | 0.879994 |
| Psmd8 | -0.204368 | 0.339737 | 0.524122 |
| Bag3 | 0.101759 | 0.73451 | 0.434498 |
| Plk1 | -0.106974 | -1.77071 | -1.57279 |
| Mki67 | 0.0349282 | -0.943798 | -0.955416 |
| Dusp4 | 0.0344837 | 0.950021 | 0.787148 |
| Mt2 | 1.2444 | 5.84381 | 5.55209 |
| Mt1 | 0.670113 | 4.29873 | 3.99035 |
| Cryab | 0.0866108 | 2.39276 | 2.25394 |
| Kif23 | -0.276842 | -0.669202 | -1.08413 |
| Ptgs2 | -0.0860635 | 1.37697 | 1.07984 |
| Atp6v0b | 0.0160532 | 0.799689 | 0.610683 |
| Reep4 | -0.265866 | -0.897226 | -0.785966 |
| Nek9 | -0.0439044 | -0.319377 | -0.561472 |
| Trim59 | -0.0191622 | -0.886573 | -0.799282 |
| Ifit1 | 0.166383 | -0.31537 | -0.916535 |
| Dusp5 | 0.452751 | 1.26958 | 1.13513 |
| Lars2 | 0.531132 | 0.79491 | 0.629369 |
| Ccl7 | 0.364517 | -0.0982761 | -0.851825 |
| Ythdc1 | -0.374073 | 0.985966 | 0.285639 |
| Gadd45a | -0.114842 | 0.778188 | 0.645523 |
| Tubb4b | -0.0676212 | -0.755103 | -0.594452 |
| Clic4 | 0.046338 | 0.472135 | 0.442711 |
| Tap1 | -0.143992 | -0.542792 | -1.39373 |
| Serpine1 | 0.0145349 | 1.78957 | 1.7499 |
| Klf10 | 0.439874 | 1.13619 | 0.786961 |
| Ckap2 | 0.3752 | -1.07104 | -0.797232 |
| Ncapd2 | -0.307311 | -1.34005 | -1.2613 |
| Txnip | -0.108647 | -0.838853 | -1.09412 |
| Akap12 | -0.318115 | -0.73328 | -0.740434 |
| Thbs1 | -0.120046 | -0.415221 | -0.469518 |
| Fam102b | -0.32701 | -0.511327 | -0.600008 |
| Ppp1r15a | 0.573373 | 1.85377 | 1.28844 |
| Blvrb | 0.298991 | 1.50597 | 1.77386 |
| Pvr | 0.598172 | 0.620153 | 1.15673 |
| Cirh1a | 0.0745437 | 0.413743 | 0.589867 |
| Maff | 0.208446 | 1.11259 | 0.893452 |
| Zfp36 | 0.324221 | 0.874281 | 0.571677 |
| Sdpr | -0.324479 | -0.682488 | -0.450342 |
| Plaur | 0.285172 | 0.939048 | 1.23978 |
| Irgm1 | 0.0707158 | -0.238873 | -1.14298 |
| Ckap2l | -0.11511 | -0.921397 | -1.00459 |
| Slc25a44 | 0.409909 | 1.18505 | 0.985391 |
| Ftl1 | 0.254191 | 0.878685 | 0.71623 |
| Gja1 | -0.0701084 | -0.631452 | -0.479622 |
| Ywhag | 0.154087 | 0.613517 | 0.496633 |
| Jun | 0.180297 | 0.651914 | 0.51335 |
| Junb | 0.260162 | 0.719289 | 0.356634 |
| Gas1 | 0.302091 | -1.03767 | -1.30935 |
| Zfand2a | -0.168718 | 1.16373 | 1.1013 |
| Nt5dc3 | 0.326637 | 0.907234 | 0.912352 |
| Ado | -0.476097 | -0.446913 | -0.984279 |
| Palld | 0.556988 | 0.547316 | 0.609675 |
| Espl1 | -0.0768382 | -1.03532 | -0.754481 |
| Tmed9 | 0.34289 | 0.500688 | 0.641704 |
| Ftl1-ps1 | 0.145078 | 0.987833 | 0.791471 |
| Gm10184 | 0.0987362 | -1.45939 | -0.636664 |
| Flnc | 0.168569 | 1.43176 | 1.45488 |
| Ptges3 | 0.105071 | 0.435813 | 0.586495 |
| Ifit3 | 0.161077 | -0.296905 | -1.264 |
| Ctdsp2 | -0.0125844 | -0.490488 | -0.61135 |
| Prnp | 0.0945729 | 0.625417 | 0.533125 |
| Kifc1 | -0.403478 | -1.7009 | -1.54638 |
| Slc48a1 | 0.133396 | 1.3211 | 1.24651 |
| Gm5844 | 0.324679 | 1.46837 | 1.25648 |
| Gm12346 | -0.195467 | 1.30713 | 1.12814 |
| Gm17046 | 0.505019 | 1.27318 | 1.7183 |
| Gm8355 | -0.287156 | 0.893697 | 0.500816 |
| Gm5456 | 0.445441 | 1.21676 | 1.49577 |
| Syne1 | 0.471495 | 0.413027 | 0.993066 |
| H1f0 | 0.0643345 | -0.475685 | -0.341142 |
| C920009B18Rik | -0.382452 | 5.46273 | 6.92147 |
| Gm26917 | 0.130098 | 1.08715 | 0.902392 |
| Gm15459 | 0.009872 | 1.00199 | 0.740634 |
