# Supplementary material for: Core–shell nanoparticles suppress metastasis and modify the tumour-supportive activity of cancer-associated fibroblasts
Source: J Nanobiotechnology. 2020 Jan 21;18:18. doi: 10.1186/s12951-020-0576-x (PMC6974972; doi:10.1186/s12951-020-0576-x)
Supplement: Supplementary file 16 — Additional file 16. Kaplan–Meier plots of low and high expression levels of Ptn, Adamts5 and Thbs2 genes in breast cancer patients. [file 12951_2020_576_MOESM16_ESM.docx]

**Additional File 16.**
