# Supplementary material for: Core–shell nanoparticles suppress metastasis and modify the tumour-supportive activity of cancer-associated fibroblasts
Source: J Nanobiotechnology. 2020 Jan 21;18:18. doi: 10.1186/s12951-020-0576-x (PMC6974972; doi:10.1186/s12951-020-0576-x)
Supplement: Supplementary file 17 — Additional file 17. TCGA expression data of selected genes in normal and matching cancerous breast cancer tissues. [file 12951_2020_576_MOESM17_ESM.docx]

**Additional File 17.**
